# Robots and Minimal, Physics‐Informed Features: A Hybrid Framework for Enzyme Catalysis

**DOI:** 10.1002/advs.77474

**Published:** 2026-09-11

**Authors:** Natalia Onishchenko, Eric S. Larsen, Govind Paneru, Kisung Lee, Diana V. Kolygina, Yankai Jia, Elizabeth Maria Clarissa, Wai‐Shing Wong, Bartosz A. Grzybowski

**Affiliations:** ^1^ Center For Algorithmic and Robotized Synthesis Institute For Basic Science Ulsan Republic of Korea; ^2^ Department of Physics Ulsan National Institute of Science and Technology Ulsan Republic of Korea; ^3^ Department of Biomedical Engineering Ulsan National Institute of Science and Technology Ulsan Republic of Korea; ^4^ Department of Chemistry Ulsan National Institute of Science and Technology Ulsan Republic of Korea

**Keywords:** biocatalysis, enzyme models, machine learning, robotics, substrate scope

## Abstract

The utility of enzymes in organic synthesis is constrained by the limited ability to predict the scope of small‐molecule substrates that a given enzyme can act upon. The problem has proven challenging to both classical computational‐chemistry approaches and to modern Machine Learning (ML) methods, the latter suffering from the combination of data scarcity (including all‐important negative examples) and the inaccuracy of chemoinformatic vectorization schemes. The current work addresses both problems, deploying affordable chemical robotics to curate a structurally diverse set of both active and inactive substrates, and then using this data to develop a physics‐grounded ML model for substrate scope prediction. This model uses only a handful of features to learn the proper balance between steric and electronic factors even from small datasets. It maintains useful predictive power on external enzyme families and shows improved out‐of‐distribution performance compared with descriptor‐heavy state‐of‐the‐art ML algorithms.

## Graphical abstract

Robotic experimentation and physics‐informed machine learning combine to predict enzyme substrate scope. With a handful of interpretable features derived from docking and quantum mechanics calculations, our model rivals descriptor‐heavy AI approaches and extrapolates to unseen substrates and enzyme classes.

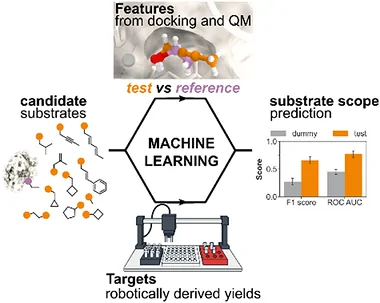

## 1 Introduction

Due to their inherent “greenness” and ability to catalyze various organic transformations—often on complex substrates and with high enantioselectivity—enzymes are highly valued in modern synthesis, across both academic [1] and industrial [2, 3] domains. Yet, despite several high‐profile successes (e.g., sitagliptin [4], atorvastatin [5], pregabalin [6], sacubitril [7], tenofovir [8]), a recent survey [9] revealed that enzymes remain underutilized. Their deployment still relies heavily on serendipitous “hits” from empirical enzyme–substrate screens, rather than systematic, predictive frameworks for substrate scope. This lack of predictability stems from the acute sensitivity of enzymatic activity to subtle structural variations in substrates, which can dramatically alter interactions within the enzyme's active site. Not surprisingly, this challenge has spurred considerable theoretical effort [10, 11, 12, 13, 14, 15, 16], including various Machine Learning (ML) models [11, 16, 17, 18, 19, 20, 21, 22, 23, 24, 25, 26] which encode substrate and enzyme structures with large sets of chemoinformatic descriptors. These models sometimes report very high accuracies [21, 23, 24] under cross‐validation but are also known to fail when confronted with ligands (and to a lesser extent enzymes) unseen during training [22, 23].

**FIGURE 1:**
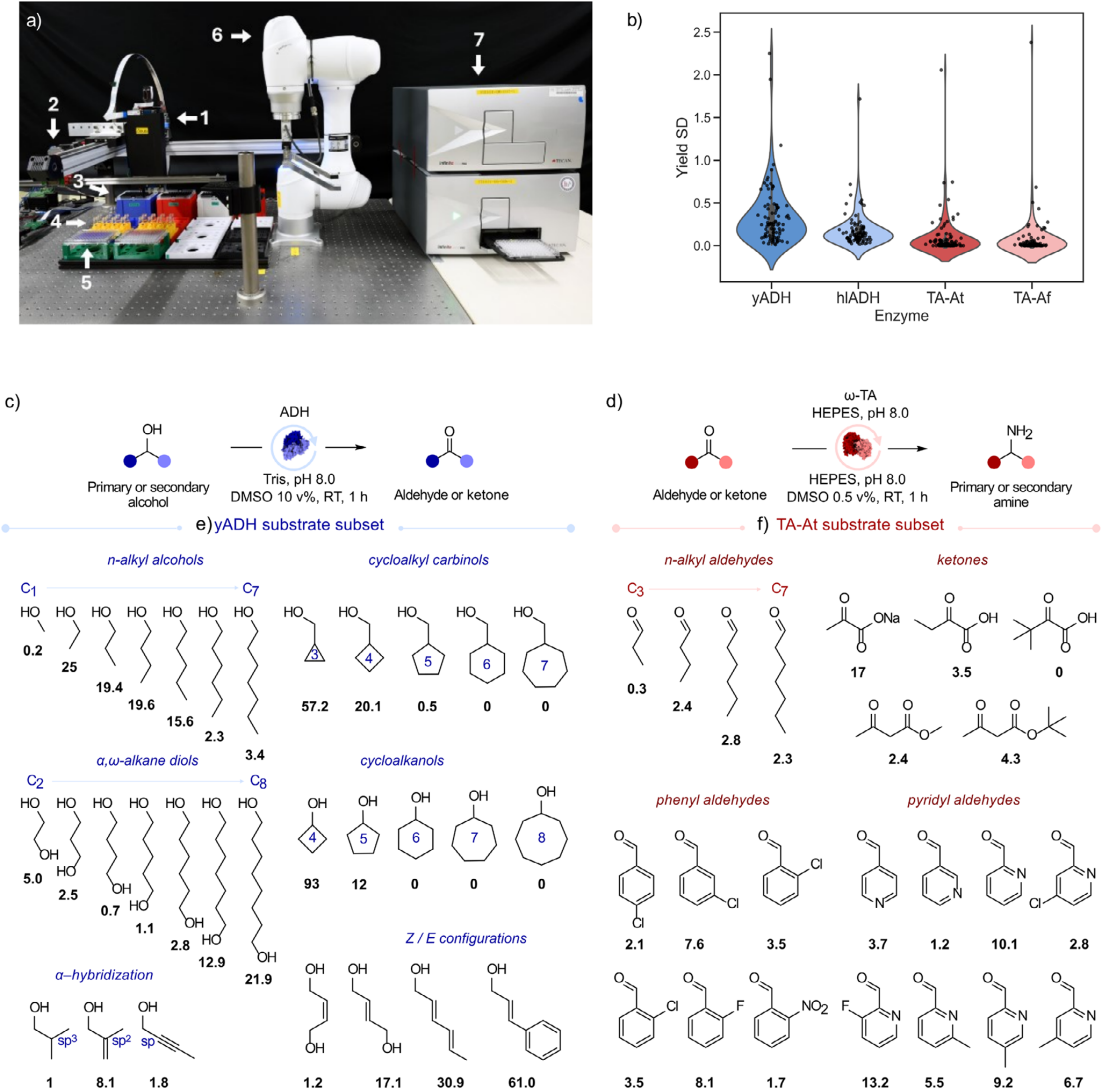
(a) An in‐house‐built Robowski platform for reaction preparation and characterization. This platform is designed to prepare reaction mixtures over 96‐well plates, transfer these plates to the plate reader, acquire spectra, and use custom computer codes to interpret the results. For replication blueprints, see Ref. [32]; for computer codes used here, see Ref. [35]. The main components shown in this photograph are (1) liquid handling module (Zeus pipette channel, Hamilton Company, Reno, NV), (2) horizontal two‐axis gantry (FPB series, Fuyu Motion, China), (3) racks of pipette tips, (4) racks with vials for stock solutions, (5) 96‐well plates, (6) robotic arm (A0509, Doosan Robotics, South Korea) for well‐plate transfer, and (7) UV‐Vis / fluorescence plate reader (M200, Tecan, Austria). See also Movie S1. (b) Yield measurement error distribution for the four enzymes tested in this work (reported in percent to total expected yield): yeast and horse liver alcohol dehydrogenases (yADH and hlADH) and ω‐transaminases from *A. terreus* and *A. fumigatus* (TA‐At and TA‐Af). Errors are based on triplicate experiments for all substrate–enzyme pairs. (c, d) Schemes of the enzymatic reactions and examples of reaction outcomes. (c) Yeast and horse liver alcohol dehydrogenases (ADHs) (EC 1.1.1.1) catalyze oxidation of primary or secondary alcohols to aldehydes or ketones under basic conditions. In this reaction, the NAD^+^ cofactor is being reduced to NADH. (d) Recombinant ω‐transaminases (from *A. terreus*, TA‐At, EC 2.6.1.18, and *A. fumigatus*, TA‐Af, EC 2.6.1.42) are used to make enantiopure amines from prochiral aldehydes or ketones. Examples of reaction outcomes (% yields) are listed below the compounds in (e) for yADH and in (f) for TA‐At. For discussion of the most interesting cases, see main text. For outcomes for all substrates and enzymes, see Figure S5.

A major obstacle to improving algorithms for substrate scope prediction is the scarcity of relevant and high‐quality data. In widely used databases such as BRENDA 2024.1 [27] (www.brenda‐enzymes.org), the average enzyme is associated with only three substrates, and 99% of enzymes have fewer than 24 substrates. Moreover, negative examples—substrates that do not react—are rarely reported. Such sparse and biased coverage of the structural space is insufficient given the aforementioned sensitivity of reaction outcomes to minor structural variations (see, e.g., Figure 1e,f). The situation is not readily remedied by data augmentation techniques, which can boost performance on the test–train splits [22, 23, 24] but, as we shall see later in this work, do not help with out‐of‐distribution predictions.

Here, to address these limitations, we harness the synergy between chemical robotics and model development. First, using a custom‐built, cost‐effective robotic platform, we quantify enzymatic reaction yields across a structurally diverse set of both active and inactive substrates. This approach generates a dataset of 374 enzyme–substrate pairs (across four enzymes), each analyzed in triplicate. We then use this unique dataset to develop and validate a “hybrid” model that combines ML with physics‐based descriptors designed to capture steric and electronic differences between a substrate of interest and the enzyme's native substrate (serving as reference for catalytic activity). Despite relying on only a handful of features, this model achieves accuracies on par with state‐of‐the‐art ML algorithms built on large, (un)constrained chemo(bio)informatic inputs. It generalizes well across several enzyme classes and extrapolates with comparable accuracy to curated literature datasets. Models like this one can be helpful in prescreening substrates amenable to enzymatic catalysis, or as part of synthesis planning programs, in which inclusion of enzymatic transformations is one of the desirable features but currently relies on inadequate, similarity‐based metrics [28, 29, 30, 31].

## 2 Results and Discussion

### 2.1 Robotic System

Our initial objective was to establish a robust experimental dataset comprising high substrate‐to‐enzyme ratios (∼100), with all reactions conducted under standardized conditions and with yield measurements rigorously validated. For these studies, we adapted our in‐house‐built Robowski platform (Figure 1a and Movie S1), which we had previously used for the reconstruction of large “hyperspaces” or conditions for diverse chemical reactions [32, 33, 34]. This robotic system uses relatively inexpensive parts (in the basic configuration, ∼$25 K plus a standard plate reader), offers 1%–2% pipetting accuracy, and is easy to replicate using blueprints detailed in Ref. [32]. Here, the reaction preparation module (over standard 96‐well plates) was interfaced with an Infinite 200 PRO (Tecan, Austria) microplate reader for automatically assaying the reaction outcomes. Optionally but not essentially, a robotic arm (A0509, Doosan Robotics, South Korea) was used for the transfer of plates to and from the plate reader. The cost per screened substrate is estimated to be below $7, including low‐protein 96‐well plates, conductive pipette tips, and buffer components. After assay conditions are optimized, the platform can screen up to approximately 300 substrates per workday (8–10 h). All reactions were performed in triplicate and were allowed to proceed for 50–60 min. Reaction yields (rather than, e.g., *K*_M_ values) were quantified as more relevant to synthetic chemistry and simpler for screening. We should note, however, that endpoint yields are influenced by multiple factors determined by the assay conditions (like substrate solubility, product inhibition, equilibrium position, cofactor availability, enzyme stability, and assay interference) and should only be used as an approximation of catalytic efficiency. These yields were determined by assays detailed in the Supplementary Information Experimental Details, Section 2, and using appropriate calibration curves of the reaction products and data processing codes deposited online [35]. In both assays, yields are reported with respect to limiting reagents, and we use them as a measure of enzyme activity toward (the nonlimiting) substrate. For all enzyme types, standard deviation of yields was, on average, below 1% yield (see violin plots in Figure 1b).

### 2.2 Choice of Enzymes and Substrates

We chose four commercially available and relatively inexpensive enzymes belonging to two families: (1) Two medium‐chain alcohol dehydrogenases (horse liver dimer, hlADH, and yeast tetramer [36] yADH; EC 1.1.1.1) that catalyze reversible oxidation of alcohols to aldehydes or ketones (Figure 1c); and (2) Two recombinant ω‐transaminases (from *Aspergillus terreus*, TA‐At, EC 2.6.1.18, and *A. fumigatus*, TA‐Af, EC 2.6.1.42) that produce enantiopure amines from prochiral aldehydes or ketones (Figure 1d). The existing literature provides only a limited set of substrates for these enzymes. yADH is reported to be active with respect to primary alcohols (in particular, ethanol), and UniProt (yADH UniProt ID P00330) reports *K*_M_ values for 9 primary and 3 secondary alcohols of varying chain length. hlADH (UniProt ID P00327) has a broader substrate specificity [37], with BRENDA reporting 26 alcohol substrates with *K*_M_ values, including branched and relatively long‐chain alcohols. For TA‐At (UniProt ID Q0C8G1), BRENDA reports only two carbonyl substrates and none for TA‐Af. Substrate scope of transaminases has been the subject of extensive research of synthetic chemists, with earlier works summarized by Calvelage and co‐authors [38]. Their dataset included 62 unique substrates for TA‐At and 12 for TA‐Af, however, the data were derived from overall 10 papers, thus obtained under varying conditions.

To broaden this substrate scope, we searched the PubChem database for primary and secondary alcohols, as well as aldehydes and ketones. We then downselected the results to those that had MW < 400 and were commercially available and relatively inexpensive (up to ∼$100/g). We ultimately purchased 258 entities and tested them for solubility in the assay buffers (see Experimental Details, Section 2). This left 89 compounds as potential substrates for ADHs and 98 as substrates for TAs; both sets are detailed in Figures S1 and S2. We note that although, by construction, substrates within each enzyme class shared the same or very similar reactive headgroups, the remaining fragments of these molecules were relatively diverse. Specifically, when clustering the substrates within each subgroup (ADH and TA substrates) using the k‐medoids algorithm, there was no clear “elbow” in any of the plots of the k‐medoid scores against the number of clusters (Figures S3 and S4). Such behavior is indicative of poor data clusterization and hence diversity. Calculated average silhouette coefficients [39] for the clusters for all compounds were also consistently low (Figures S3 and S4).

In total, 374 enzyme–substrate pairs were screened. Within this collection, 42 out of 89 yADH substrates gave yields >1%, whereas for hlADH, this proportion was 52 out of 89 under the standardized assay conditions (see Figure S5a and Table S1 for specific values). To access a wider range of substrates, reactions were run in the presence of 10% DMSO, which did not affect enzyme activity. To shift the reaction toward the products, excess NAD^+^ was used. As a reference, the yields of the “native” ethanol substrate were 25 ± 2% for yADH and 13.5 ± 0.7% for hlADH. For TA‐At, 28 out of 98 substrates screened had >1% conversion rates, and for TA‐Af, 16 out of 98 (Figure S5b). The reaction requires two substrates, an amine donor and amine acceptor, and a cofactor (pyridoxal phosphate, PLP). In the assay, the amine acceptors were varied while all reactions shared the same amine donor, (R)‐1‐(6‐methoxynaphthalen‐2‐yl)ethanamine (MNEA), and were run in the presence of external PLP. The conversion rates of the native sodium pyruvate substrate were 17 ± 2% for TA‐At and 14.7 ± 0.7% for TA‐Af. Foreshadowing our discussion later in the text, substrates with yields >1% were classified as “active” and those with yields ≤1% as “inactive”. This threshold was chosen for three reasons: (i) the lowest value for which the yields could be determined reliably was below 1% (*p* < 0.05 in single‐tail equal variance t‐tests between substrate‐free control and samples); (ii) Although such yields are not synthetically practical, they can provide a starting point for productive enzyme evolution campaigns, as evidenced by prior examples [4]; (iii) 1% ensures a relatively balanced split between actives and inactives, which is beneficial for model training (for higher yield thresholds, the set is dominated by inactives; see Figure S6a for comparison of the model performance with 1%, 3%, and 5% thresholds). Thus, hereinafter, we refer to weak hits with detectable enzymatic activity as “actives.”

### 2.3 Examples of Yield Trends, Activity Cliffs and Fallacy of Similarity Metrics

The yields for all enzyme–substrate pairs are summarized in Figure S5 (and detailed in Table S1). A subset focusing on a series of homologous substrates is showcased in Figure 1e,f. Starting with yADH in Figure 1e, we note that even for simple *n*‐alkyl alcohols, the trend is nonlinear, with yield increasing sharply from C_1_ to C_2_ but then decreasing for C_6_ and C_7_. For the family of α,ω‐alkane diols, the yields are low up to C_6_ but then increase sharply for C_7_. By contrast, for cycloalkyl carbinols and cycloalkanols, the yields drop sharply for larger rings. Other examples illustrate strong effects of hybridization of atoms away from the reaction center, including cases when a difference of *Z* vs. *E* isomerization of a single double bond can result in a 1.2 ± 0.3% vs. 17.1 ± 0.2% difference in yield. For TA‐At substrates in Figure 1f, variations in chain length have little impact on reactivity, but there are strong effects due to substituents at the α‐carbon or at proximal aromatic rings. Overall, such examples give us a flavor of why prediction of substrate activity vs. inactivity is a difficult problem, sensitive to minor structural differences and reminiscent of the “activity cliffs” [40, 41] often observed in drug discovery. They also remind us that simple metrics of molecular similarity (e.g., Tanimoto) used in many enzymatic synthesis planning programs [30, 31] might be insufficient—not because the metric itself is flawed, but because the underlying 2D representations it compares do not explicitly encode key determinants of enzymatic turnover (e.g., 3D steric complementarity/binding pose and local electronic effects). Thus, it is not generally valid to assume that an algorithm‐proposed substrate will be enzymatically active simply because it is similar to some other substrate of known activity towards this enzyme.

### 2.4 Physics‐Based Model Features

The above results suggest that ML based on the representations of protein sequence and on fingerprint or graph descriptors of the small‐molecule ligands may not fully capture how these ligands interact with the complex, macromolecular environment of the active site. Indeed, highly nonlinear trends within series of homologous compounds (cf. Section 2.3 and Figure 1e,f) all point to the importance of specific conformations (defining enzyme–substrate interactions and, consequently, substrate's orientation within the active site), as well as the electronic effects (altering the reactivity of the “headgroup” undergoing specific transformation).

To capture the conformational aspects, we decided to base our model on familiar docking [42, 43, 44, 45]. Here, we deployed a popular and freely available AutoDock4, mostly because its AutoDock4 Zn version is well suited [46] to account for the interactions of the substrate with the Zn atom present in ADHs. To select the docked conformation of a substrate, we took into account minimum binding energy and pose consistency. Then for the electronic effects, we performed quantum‐mechanical calculations (DFT using the B3LYP functional and def2TZVP basis set) (see Supporting Information. for Experimental Details).

Naturally, the limitations of the docking algorithms in terms of the accuracy of the poses and absolute values of the binding scores/energies are well known [47, 48]. In this context, our goal was not to make absolute‐value predictions but rather to look into the differences in the poses and energies of the enzyme's known reference substrate *S_ref_* and other substrates *S_i_* sharing the same reactive “headgroup.” The native substrate is often the preferred choice once identified, as enzymes have typically evolved to catalyze reactions involving it with high efficiency. However, we also verified that a productive substrate other than the native one can be used as a reference (Figure S7b,c).

With these preliminaries, we hypothesized that some substrate *S_i_* can be active (i) if its headgroup is able to assume an orientation at the reaction center similar to that of the reference substrate *S_ref_*, and (ii) if the electronic properties of the headgroups of *S_i_* and *S_ref_* are also similar. Fulfillment of (i) and (ii) does not ensure catalysis but can be viewed as a precondition for catalysis to occur—differently put, if *S_i_* cannot even assume the orientation compatible with productive binding within the active site, it is unlikely to undergo catalysis.

**FIGURE 2:**
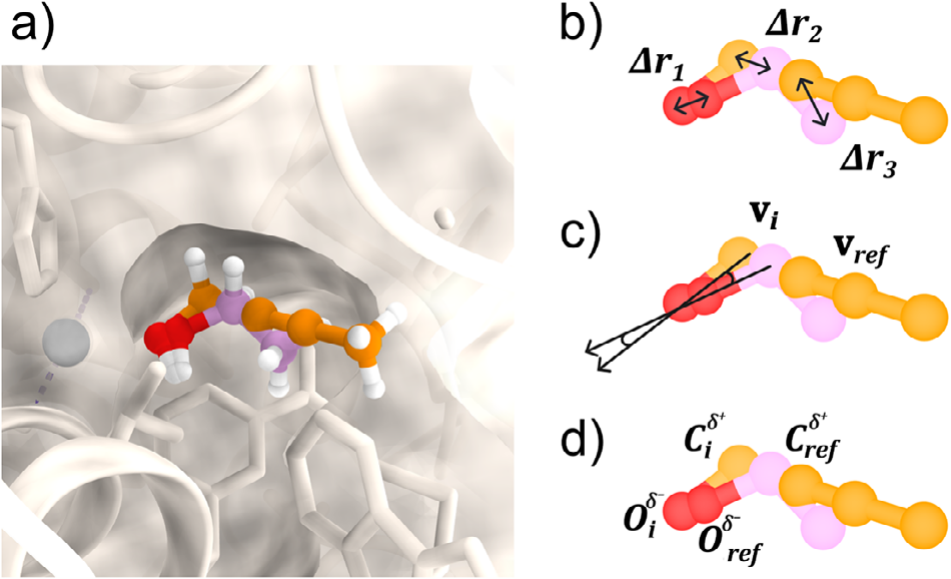
Illustration of model's key features. Scheme in (a) shows superposition of the native ethanol substrate (*S_ref_*, carbon atoms colored in purple) and 2‐butyn‐1‐ol substrate (*S_i_*, carbon atoms colored in orange), both docked into the active site of yADH. Schemes (b)–(d) illustrate the metrics defining the differences in the docking poses or electronic properties of the two substrates: (b) differences Δ*r_j_* in the positions of the corresponding heavy atoms of the reacting headgroup on *S_i_* and *S_ref_*; (c) the angle γ between the vectors v defining corresponding bonds within the headgroup of the two substrates (here, the C─O bond); (d) the differences Δ*q_j_* in NBO charges on carbon and oxygen atoms of the bond undergoing transformation.

With these considerations and with reference to Figure 2, the following eight features of any substrate *S_i_* were considered (for further discussion of feature selection, see later in the text and Section S4):

(1) The root‐mean‐square deviation (RMSD) between the coordinates of the corresponding heavy atoms in the headgroups of *S_i_* and *S_ref_* substrates, defined as $\sqrt{\frac{1}{N}\sum_{j=1}^{N}|r_{j,i}-r_{j,\mathrm{ref}}{|}^{2}}$, where *N* is the number of atoms considered in the substrate, and *r_j_* ‘s are position vectors of corresponding atoms *j* in substrates *S_i_* and *S_ref_* (Figure 2b).

(2) Angular deviation between the corresponding headgroup bond(s) *k* in *S_i_* and *S_ref_* and defined as $\gamma_{k}=\arccos\left(\frac{v_{k,i\;}•\;v_{k,ref}}{|v_{k,i}\left|\right|v_{k,ref}|}\right)$. Here, we consider only one bond for each enzyme type (C─O in ADHs and C═O in TAs; Figure 2c) and thus use only one bond vector $v_{C\to O\;}=r_{O}\;-r_{C}$.

(3) Binding energy difference between *S_i_* and *S_ref_*:Δ*E_i_* = *E_ref_*  − *E_i_*, where *E_ref_* and *E_i_* are the binding energies of the lowest‐energy docked poses of *S_ref_* and *S_i_*, respectively.

(4, 5) Difference in the QM‐calculated Natural Bond Orbital (NBO) partial charges on the corresponding *j* atoms of *S_i_* and *S_ref_*,Δ*q*_*j*,*i*_ = *q*_*j*,*ref*_  − *q*_*j*,*i*_. Here, we consider only the headgroup C and O atoms (Figure 2d).

(6–8) Difference in the QM‐calculated HOMO and LUMO energies of S_i_ and S_ref_ defined as $\Delta E_{i}^{HOMO(LUMO)}=E_{ref}^{HOMO(LUMO)}\;-E_{i}^{HOMO(LUMO)}$, where $E_{ref}^{HOMO(LUMO)}$ and $E_{i}^{HOMO(LUMO)}$ are the HOMO (LUMO) energies of S_ref_ and S_i_, respectively. Additionally, the difference in HOMO–LUMO gap was calculated as $\Delta E_{gap,\;i}=\Delta E_{i}^{LUMO}-\;\Delta E_{i}^{HOMO}$.

Features #1 and #2 quantify if the headgroup of the docked substrate *S_i_* is close to and not rotated adversely with respect to the productive orientation of the reference substrate *S_ref_*—if *S_i_* cannot reach this orientation (e.g., *S_i_* is too bulky or other interactions with the active site prevent this orientation), the desired biotransformation will be hampered. Feature #3 quantifies how favorably *S_i_* binds compared to *S_ref_*. Even if proper orientation and favorable binding can be reached, electronic factors captured by #4–8 may still be important to determine the reaction's un/feasibility. In other words, the approach captures necessary preconditions rather than catalytic competence per se.

This said, we also considered the features #3–6 (binding energy, partial charges and HOMO energy difference) of the reaction products, with the rationale that deviation of these product feature values from the substrate feature values should be advantageous for the product to dissociate from the active site. However, inclusion of these product parameters did not improve the model's performance (Figure S7a) and will not be considered in the rest of our discussion.

### 2.5 Model Development

**FIGURE 3:**
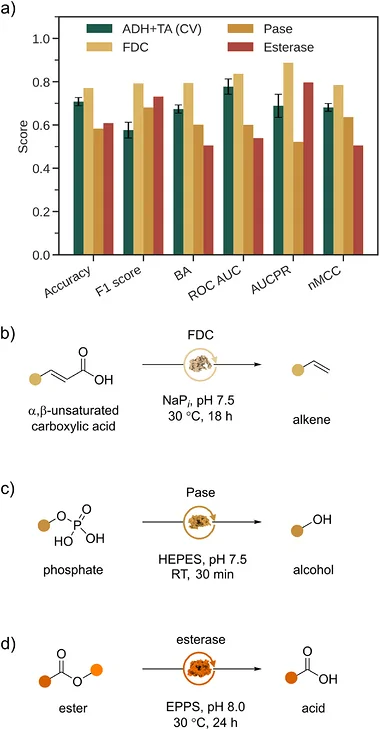
(a) Performance of the random forests (RF) model trained on 4 enzymes (yADH, hlADH, TA‐At, and TA‐Af). Dark green bars are 5‐fold CV performance metrics. BA, balanced accuracy; ROC AUC, receiver operating characteristic curve area under the curve; AUCPR, area under the precision–recall curve; and nMCC, normalized Matthew's coefficient. Pale orange bars quantify the performance of the RF model on the external FDC dataset in Ref. [49] (FDCs; EC 4.1.1.61; see SI Experimental details), whereas dark orange bars, on the phosphatase (Pase) literature dataset adopted from Goldman et al. [19]. (b) FDCs catalyze decarboxylation of α,β‐unsaturated carboxylic acids to alkenes. (c) *Escherichia coli* phosphatase catalyzes the dephosphorylation of alpha‐D‐glucose 1‐phosphate (G1P) and, to a lesser extent, of other sugar phosphates (EC 3.1.3.10) [50]. (d) Ester hydrolase (esterase) CalB from *Pseudozyma aphidis* (EC 3.1.1.3) catalyzes hydrolysis of esters in Ref. [51].

We had five enzyme family datasets at our disposal: two experimental datasets (i.e., all substrates for two ADHs and all substrates for two TAs) and three external datasets (see below) to evaluate the generalizability of the developed docking/QM features. The first external dataset contained 48 substrates (30 actives and 18 inactives) of fungal ferulic acid decarboxylases (FDCs; EC 4.1.1.61) catalyzing decarboxylation of α,β‐unsaturated carboxylic acids to alkenes (Figure 3b) [49]. This set was chosen because it reported yields (rather than *K*_M_ constants) and because it reported both actives and inactives, the latter with zero yields. Two other external datasets were extracted from larger datasets of phosphatase (Pase) and esterase activity (Figure 3c,d) often used to test models predicting enzyme substrate specificity, which makes them a useful comparison [19, 50, 51]. Selected Pase (EC 3.1.3.10) had 52 positives and 56 negatives according to the reactivity threshold chosen by the dataset authors [50]. As for the esterase dataset [51], after data filtering (Supporting Information Section), it contained 63 positives and 25 negatives.

Four model architectures were implemented: Logistic Regression, LR, decision trees, DT, random forests, RF, and XGBoost, XGB. The first step of model development involved selection of the optimal architecture and feature set. The five enzyme family datasets were split into exhaustive combinations of 2 training (TRAIN), 1 validation (VAL), and 2 test (TEST) family datasets.

Only TRAIN and VAL datasets were used for model architecture and feature selection. For each enzyme family as a VAL set, there were six possible TRAIN datasets (e.g., ADH + TA → FDC; ADH + Pase → FDC; Pase + TA → FDC; ADH + Esterase → FDC; TA + Esterase → FDC; Pase + Esterase → FDC). These six models were trained with separate hyperparameter optimization. Feature ablation (3–8 features) was performed on 30 models (6 TRAIN combinations x 5 VAL dataset families). For each VAL dataset, prediction scores per substrate were averaged across these six data splits to calculate family‐wise validation BA. Then, an average validation BA over all five enzyme families (each as a validation) was calculated. These validation BAs were in turn averaged over model architectures for each feature combination and ranked to select the best feature set (RMSD, angular deviation, and HOMO–LUMO gap; see Figure S8). When the feature set was fixed, RF was the highest‐performing architecture (Figure S9).

After the RF architecture and the three‐feature set were selected, we used the model to obtain test set predictions on the TEST enzyme families that were not seen by the model for each TRAIN/VAL combination. Remembering that the datasets are imbalanced, we considered appropriate metrics (F1 score, balanced accuracy (BA), area under precision–recall curve (AUCPR), receiver operating characteristic curve area under the curve (ROC AUC), and normalized Matthew's correlation coefficient (nMCC)). In the final model for each enzyme family, probabilities for each test substrate were averaged over all TRAIN/VAL models (12 combinations of 3 enzymes out of 4 possible). The test BA value averaged over 5 enzyme families was 0.67; see Figure S10 for a scheme of metrics aggregation per enzyme (panel a) and per enzyme performance of the ensemble model (panel b). Figure 3a quantifies CV metrics for the RF model with the three best‐performing features trained only on the TA and ADH datasets produced in this work and its extrapolation metrics on unseen datasets. Notably, the model captures whether a substrate can react, not how well. The accuracies of this binary approach remain similar when the active/inactive cutoff is moved from 1 to 3% to 5% across all five families (Figure S6).

**FIGURE 4:**
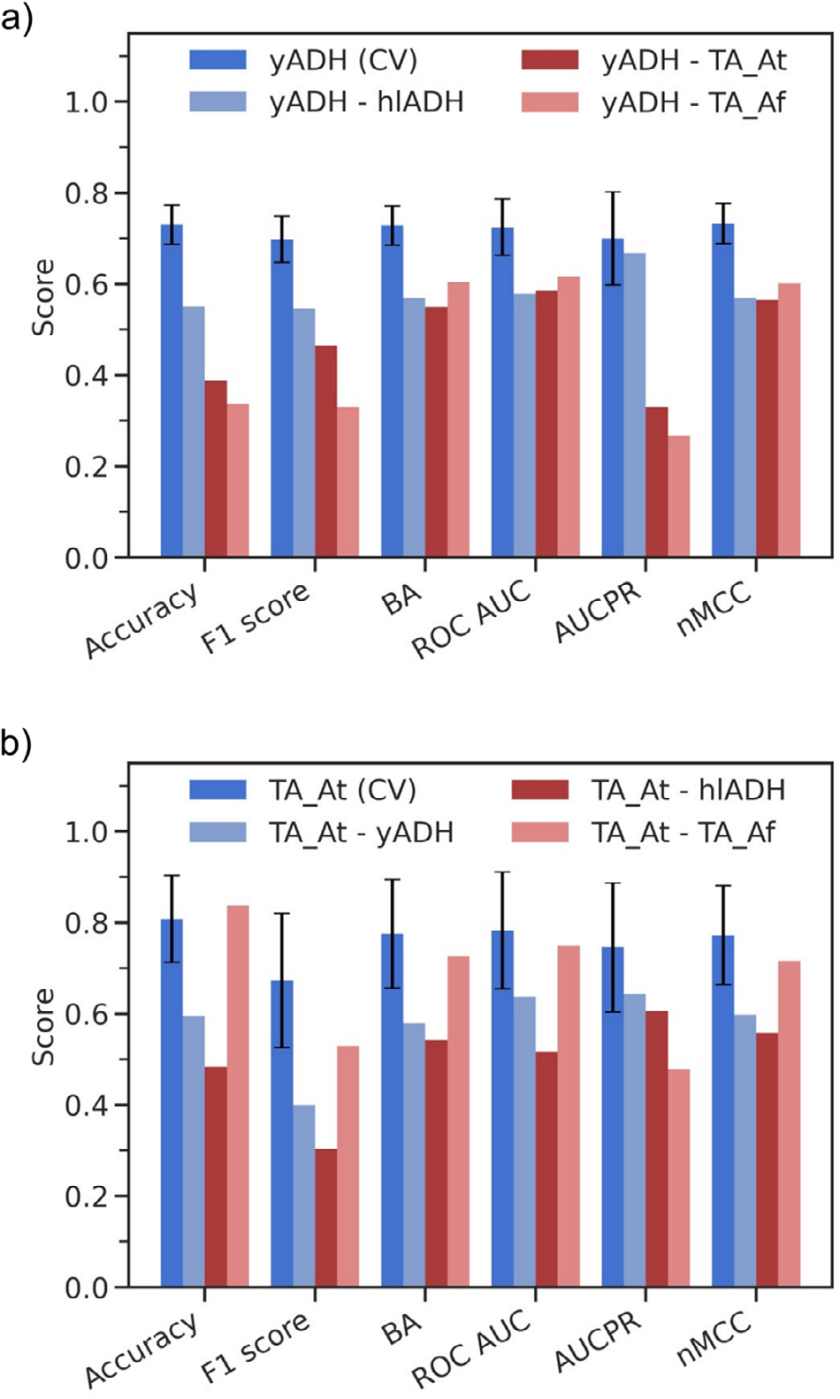
Performance of models trained on only one protein. Performance of the random forests (RF) model trained and validated (under 5‐fold CV) on only one protein and then tested on the remaining three proteins. (a) RF trained and validated on yADH data and tested on unseen data for hlADH, TA‐At, or TA‐Af and (b) trained and validated on TA‐At data and tested on unseen data for TA‐Af, yADH, or hlADH. Error bars indicate the standard deviation of the metric from 5‐fold CV. See Figure S10 for analogous results for models trained on hlADH and TA‐Af.

We note that the RF model trained on the data of two enzymes, yADH and TA‐At, also extrapolated well to unseen datasets [19] Figure S8b). We then raised the bar even further and trained the RF model on only one enzyme–substrate subset and attempted prediction on the remaining three subsets. The model transferred relatively well to the proteins of the same class (e.g., yADH to hlADH in Figure 4a or TA‐At to TA‐Af in Figure 4b). Surprisingly, in some cases it also transferred well across classes.

**FIGURE 5:**
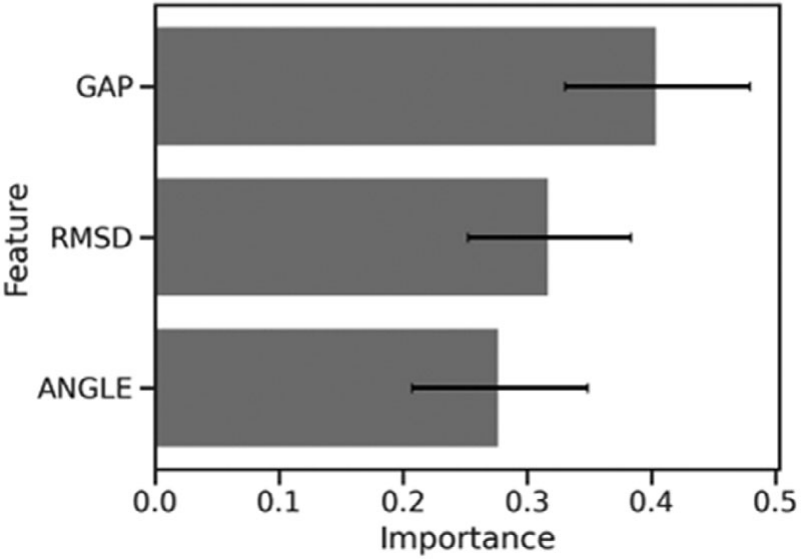
SHAP (SHapley Additive exPlanations) values obtained with TreeExplainer for Random Forest models using three features for an exhaustive set of combinations of training, validation, and testing datasets (e.g., ADH + TA → FDC, Pase; ADH + FDC → TA, Pase, and so on; see Section S4 for details).

Altogether these results suggest that features proposed in this work indeed capture the physics of the problem—even if trained on small datasets, the model can get the “right” balance between the docking and electronic features and then transfer this balance successfully to unseen enzymes and substrates. In doing so, both steric (docking: RMSD, angle) and electronic (HOMO‐LUMO gap) features are important. This is evidenced by the SHAP analysis of the three‐feature‐based model across 12 cross‐family configurations (Figure 5).

### 2.6 Comparison With State‐of‐the‐Art ML Models

**FIGURE 6:**
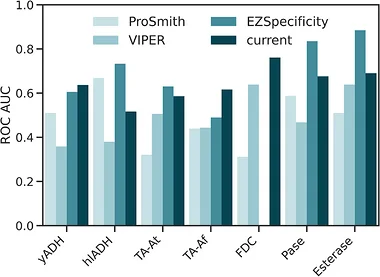
Performance of different models on external datasets in the substrate prediction task. ProSmith [23] was trained on the ESP dataset [22], VIPER was trained on the dataset prepared in [25], and EZSpecificity was trained on the ESIBank dataset [26]. Our models labeled as “current” were random forest models trained on TA‐At to test on ADHs and on yADH to test on TAs. For prediction on the external FDC [50], and phosphatase (Pase) [19, 50], and esterase [51] datasets, our random forest model trained and validated on four remaining enzyme family datasets was used. The phosphatase and esterase values for EZSpecificity and ProSmith are reported for the whole families from the Cui et al. 2025 paper [26]. In the case of EZSpecificity, performance on the two ADH and two TA datasets produced in this work was kindly provided by Prof. Huimin Zhao; data for FDC dataset were not determined for EZSpecificity.

We wondered how modern ML models would perform on our dataset—these comparative analyses are summarized in Figure 6. Initially, we tested the performance of the first general model predicting enzyme–substrate pairs developed by Kroll et al., named ProSmith [22, 23]. This transformer‐based model vectorizes both protein sequences and substrate structures, and supplements ∼13 000 experimentally validated enzyme–substrate pairs with negative samples through data augmentation, as well as phylogenetically inferred evidence. The authors have recently demonstrated this model's impressive 94.2% accuracy on test–train sets and suggested that it also should perform well for substrates similar to those seen by the model (similarity score >0.6). When tested on our dataset, ROC AUC was >0.5 only for ADHs that were seen by the ProSmith model during training. The model did not do well on the external data, including the FDC dataset we used to validate our model. This comes as no surprise since the ADH, TA, and FDC datasets contain substrates with little or no representation in Kroll's training data (only four substrates actually overlapping with the training set in the ADH and TA datasets). It has previously been pointed out that the database‐derived datasets, such as the ESP dataset used to train ProSmith, are affected by misannotations [19]. They are also heavily biased by cofactors and/or typical metabolites [25], e.g., ATP alone is a substrate in 18% of the ESP database reactions. Also, since the ESP dataset utilizes mainly native substrates as positives, it can be inherently limited in secondary substrate prediction (although the term native is arbitrary). Overall, the model appears to be operating outside its effective domain, resulting in diminished out‐of‐box performance.

The second algorithm we tested is VIPER, developed by Campbell [25] and utilizing extended‐connectivity‐fingerprint (ECFP, 1024 dimensions) featurization of small molecules and ESM‐1b representation of proteins. Like our model, VIPER is relying on dense high‐throughput enzyme substrate screens (i.e., screens that ensure a high number of substrates per enzyme). While showing ROC AUC > 0.6 in the case of the FDC dataset unseen by all models compared, VIPER performed the worst on unseen ligands from our dataset. One of the reasons could be that ECFP used in this model encode 2D topological neighborhoods and therefore lack explicit information about the molecule's three‐dimensional conformation and electronic structure. Similarly, sequence representations of enzymes (like ESM‐1b in case of VIPER) also do not carry information about 3D structures, specifically of active sites, and interaction with small molecules. This said, both ProSmith and VIPER remain valuable to make predictions for enzyme families included in the training data, and we expect their performance to improve considerably upon enlarging the training dataset [25].

Finally, we tested the most recent and comprehensive EZSpecificity model [26] trained on the largest database currently available. In addition to featurizing small molecules and enzymes separately, this model also vectorizes interactions of substrates docked into enzyme's active sites. As we expected, EZSpecificity performed better than either ProSmith or Viper, and on par with our random forest model. Both models are docking‐based and thus are active‐site aware, but the random forest model developed here uses orders‐of‐magnitude less features to train. These results support the value of using structural information explicitly, not relying solely on sequence data [52], and assigning higher weights to descriptors of the active sites (vs. rest of the protein structure). Compared to EZSpecificity, our model benefits from utilizing a small number of features, which offers interpretability.

More broadly, it should be noted that successful application of the model requires identification of a reactive headgroup, defined by the bond undergoing transformation and the adjacent atoms involved in catalysis (see Section 2.4 and Figure 2 for the example). This assignment relies on prior knowledge of the reaction mechanism and chemical intuition. Consequently, the approach is best suited to enzyme classes where a common reactive motif can be readily defined and may be less applicable to transformations that do not admit a clear reactive group representation.

While the model presented here is minimal in feature dimensionality, it requires docking and DFT calculations and thus is not minimal in the sense of the computational workflow. The inputs required for practical implementation include (i) a 3D structure of the enzyme obtained either experimentally or from fold predictions; (ii) a substrate known to be capable of productive binding mode to be appointed the reference substrate (a round of experimental screening may be required to establish such a reference if no substrate has yet been identified for the enzyme); and (iii) candidate substrate structures (see SI for required file formats). The computational requirements of the workflow as such are relatively modest: substrate poses are generated using AutoDock4, a mature and widely used docking platform, while electronic descriptors are obtained from single‐molecule DFT calculations. Hence, screening datasets of dozens to hundreds of candidate substrates can be completed within a few days on standard computational resources.

Finally, the ensemble model (see the repository at GitHub) takes docking‐derived RMSD and angular deviation and QM‐derived HOMO energy as input features and generates binary predictions, with a default threshold of 0.5. The prediction score can be used for adjusting the decision threshold by the user, as the closer the probability is to 0 or 1, the higher is the model's confidence in classifying the compound as active or inactive.

## 3 Conclusions

Overall, our study prompts two major comments that, in many ways, echo our experiences with ML vs. hybrid models in predicting outcomes of non‐enzymatic organic reactions and synthesis planning [53, 54, 55]. First, broad structural coverage and inclusion of negative data are all‐important [56]. The current methods for gathering and reporting standardized data of enzymatic affinity are not scalable, as reflected by the very sparse coverage at the high substrate‐per‐enzyme ratio. In this regard, we hope that relatively inexpensive robotic systems like Robowski can help democratize such efforts, allowing even smaller labs to perform relatively large substrate screens under standardized‐precision settings.

Second, it is important to account for both steric and electronic effects using physically realistic descriptors. For chemical reactions, we were previously able to do so [57, 58] by vectorizing the reacting partners with the hindsight (from prior mechanistic studies) of their orientations. For proteins and diverse ligands, there is no such a priori knowledge, and hence docking becomes the surrogate model in our ML pipeline. Here, we used classic docking methods, but we see much room for improvement using the modern co‐folding methods (e.g., AlphaFold 3 [59], or Boltz‐2 [60]), reporting drastically improved accuracy [59]. Showing transferability across several mechanistically distinct enzyme families, our model is promising for fixed‐enzyme substrate scope prediction and complements the state‐of‐the‐art prediction models. It could be especially beneficial in situations when enzyme and/or substrates of interest are not found in the more advanced model's training datasets.

## Author Contributions

N.O. conducted experiments, curated and analyzed data, and developed modeling approaches. E.S.L. designed and conducted experiments and analyzed data. G.P. curated data, developed computational approaches, and performed molecular docking, DFT calculations, data analysis, model development, and validation. K.L. performed ML model development and validation. D.K. performed experiments and data analysis. Y.J. developed and validated the robotic platform. E.M.C. programmed the robot‐arm and plate‐reader control software. W.S.W. developed computational approaches through contributions to machine learning feature extraction. N.O., E.S.L., G.P., and B.A.G. wrote and revised the manuscript. B.A.G. conceived and supervised the project.

## Conflicts of Interest

The authors declare no conflicts of Interest.

## Supporting information




**Supporting File 1**: advs77474‐sup‐0001‐SuppMat.pdf.

**Supporting File 2**: advs77474‐sup‐0002‐S1.mp4.

**Supporting File 3**: advs77474‐sup‐0003‐S1 legend.docx.

**Supporting File 4**: advs77474‐sup‐0004‐S1_final.xlsx.

## Data Availability

Structures of all substrates and their yields are listed in the Supporting Information. Raw data, code to dispense the solutions, collect and analyze the measurement data, dock the substrates, extract features and train models, are deposited at Zenodo [35].

## References

[1] C. K. Winkler , J. H. Schrittwieser , and W. Kroutil , “Power of Biocatalysis for Organic Synthesis,” ACS Central Science 7, no. 1 (2021): 55–71, 10.1021/acscentsci.0c01496. 33532569 PMC7844857

[2] S. P. France , R. D. Lewis , and C. A. Martinez , “The Evolving Nature of Biocatalysis in Pharmaceutical Research and Development,” JACS Au 3, no. 3 (2023): 715–735, 10.1021/jacsau.2c00712. 37006753 PMC10052283

[3] M. T. Reetz , G. Qu , and Z. Sun , “Engineered Enzymes for the Synthesis of Pharmaceuticals and Other High‐Value Products,” Nature Synthesis 3, no. 1 (2024): 19–32, 10.1038/s44160-023-00417-0.

[4] C. K. Savile , J. M. Janey , E. C. Mundorff , et al., “Biocatalytic Asymmetric Synthesis of Chiral Amines from Ketones Applied to Sitagliptin Manufacture,” Science 329 (2010): 305–310. 20558668 10.1126/science.1188934

[5] S. K. Ma , J. Gruber , C. Davis , et al., “A Green‐By‐Design Biocatalytic Process for Atorvastatin Intermediate,” Green Chemistry 12, no. 1 (2010): 81–86, 10.1039/B919115C.

[6] S. Debarge , P. McDaid , P. O'Neill , et al., “Evaluation of Several Routes to Advanced Pregabalin Intermediates: Synthesis and Enantioselective Enzymatic Reduction Using Ene‐Reductases,” Organic Process Research & Development 18, no. 1 (2014): 109–121, 10.1021/op4002774.

[7] X. Gu , J. Zhao , L. Chen , et al., “Application of Transition‐Metal Catalysis, Biocatalysis, and Flow Chemistry as State‐of‐the‐Art Technologies in the Synthesis of LCZ696,” The Journal of Organic Chemistry 85, no. 11 (2020): 6844–6853, 10.1021/acs.joc.0c00473. 32412751

[8] B. Zdun , T. Reiter , W. Kroutil , and P. Borowiecki , “Chemoenzymatic Synthesis of Tenofovir,” The Journal of Organic Chemistry 88, no. 15 (2023): 11045–11055, 10.1021/acs.joc.3c01005. 37467462 PMC10407936

[9] F. Gallou , H. Gröger , and B. H. Lipshutz , “Status Check: Biocatalysis; It's Use with and without Chemocatalysis. How Does the Fine Chemicals Industry View this Area?,” Green Chemistry 25 (2023): 6092–6107.

[10] A. D. McDonald , S. K. Bruffy , A. T. Kasat , and A. R. Buller , “Engineering Enzyme Substrate Scope Complementarity for Promiscuous Cascade Synthesis of 1,2‐Amino Alcohols,” Angewandte Chemie International Edition 61, no. 46 (2022): 202212637, 10.1002/anie.202212637. PMC9643649 36136093

[11] S. Supekar , D. W. P. Tay , W. L. Yeo , et al., “A Machine Learning‐Guided Approach to Navigate the Substrate Activity Scope of Galactose Oxidase: Application in the Conversion of Pharmaceutically Relevant Bulky Secondary Alcohols,” ACS Catalysis 14, no. 23 (2024): 17233–17243, 10.1021/acscatal.4c04660.

[12] J. Liu , J. Tian , C. Perry , et al., “Design Principles for Site‐Selective Hydroxylation by a Rieske Oxygenase,” Nature Communications 13, no. 1 (2022): 255, 10.1038/s41467-021-27822-3. PMC8752792 35017498

[13] D. Eggerichs , N. Weindorf , H. G. Weddeling , I. M. Van der Linden , and D. Tischler , “Substrate Scope Expansion of 4‐Phenol Oxidases by Rational Enzyme Selection and Sequence‐Function Relations,” Communications Chemistry 7, no. 1 (2024): 1–9, 10.1038/s42004-024-01207-1. 38831005 PMC11148156

[14] H. Duță , A. Filip , L. C. Nagy , E. Z. A. Nagy , R. Tőtős , and L. C. Bencze , “Toolbox for the Structure‐Guided Evolution of Ferulic Acid Decarboxylase (FDC),” Scientific Reports 12 (2022): 1–11. 35232989 10.1038/s41598-022-07110-w PMC8888657

[15] M. Ohashi , C. S. Jamieson , Y. Cai , et al., “An Enzymatic Alder‐Ene Reaction,” Nature 586, no. 7827 (2020): 64–69, 10.1038/s41586-020-2743-5. 32999480 PMC7534572

[16] K. Bastard , A. A. T. Smith , C. Vergne‐Vaxelaire , et al., “Revealing the Hidden Functional Diversity of an Enzyme Family,” Nature Chemical Biology 10, no. 1 (2014): 42–49, 10.1038/nchembio.1387. 24240508

[17] D. A. Pertusi , M. E. Moura , J. G. Jeffryes , S. Prabhu , B. W. Biggs , and K. E. J. Tyo , “Predicting Novel Substrates for Enzymes With Minimal Experimental Effort With Active Learning,” Metabolic Engineering 44 (2017): 171–181, 10.1016/j.ymben.2017.09.016. 29030274 PMC7055960

[18] P. R. Neubauer , S. Pienkny , L. Wessjohann , W. Brandt , and N. Sewald , “Predicting the Substrate Scope of the Flavin‐Dependent Halogenase BRVH,” Chembiochem 21, no. 22 (2020): 3282–3288, 10.1002/cbic.202000444. 32645255 PMC7754283

[19] S. Goldman , R. Das , K. K. Yang , and C. W. Coley , “Machine Learning Modeling of Family Wide Enzyme‐Substrate Specificity Screens,” PLoS Computational Biology 18 (2022): 1009853. 10.1371/journal.pcbi.1009853 PMC8865696 35143485

[20] Y. Han , H. Zhang , Z. Zeng , Z. Liu , D. Lu , and Z. Liu , “Descriptor‐Augmented Machine Learning for Enzyme‐Chemical Interaction Predictions,” Synthetic and Systems Biotechnology 9, no. 2 (2024): 259–268, 10.1016/j.synbio.2024.02.006. 38450325 PMC10915406

[21] X. Wang , D. Quinn , T. S. Moody , and M. Huang , “ALDELE: All‐Purpose Deep Learning Toolkits for Predicting the Biocatalytic Activities of Enzymes,” Journal of Chemical Information and Modeling 64, no. 8 (2024): 3123–3139, 10.1021/acs.jcim.4c00058. 38573056 PMC11040732

[22] A. Kroll , S. Ranjan , M. K. M. Engqvist , and M. J. Lercher , “A General Model to Predict Small Molecule Substrates of Enzymes Based on Machine and Deep Learning,” Nature Communications 14, no. 1 (2023): 2787, 10.1038/s41467-023-38347-2. PMC10185530 37188731

[23] A. Kroll , S. Ranjan , and M. J. Lercher , “A Multimodal Transformer Network for Protein‐Small Molecule Interactions Enhances Predictions of Kinase Inhibition and Enzyme‐Substrate Relationships,” PLOS Computational Biology 20, no. 5 (2024): 1–23, 10.1371/journal.pcbi.1012100. PMC11142704 38768223

[24] W. Qian , X. Wang , Y. Huang , et al., “Deep Learning‐Driven Insights into Enzyme–Substrate Interaction Discovery,” Journal of Chemical Information and Modeling 65, no. 1 (2024): 187–200, 10.1021/acs.jcim.4c01801. 39721977

[25] M. Campbell , “VIPER: A General Model for Prediction of Enzyme Substrates,” BioRxiv (2024): 599972, 10.1101/2024.06.21.599972.

[26] H. Cui , Y. Su , T. J. Dean , et al., “Enzyme Specificity Prediction Using Cross‐Attention Graph Neural Networks,” Nature 647, no. 8090 (2025): 639–647, 10.1038/s41586-025-09697-2. 41061744

[27] A. Chang , L. Jeske , S. Ulbrich , et al., “BRENDA, the ELIXIR Core Data Resource in 2021: New Developments and Updates,” Nucleic Acids Research 49, no. D1 (2021): D498–D508, 10.1093/nar/gkaa1025. 33211880 PMC7779020

[28] D. Probst , M. Manica , Y. G. Nana Teukam , A. Castrogiovanni , F. Paratore , and T. Laino , “Biocatalysed Synthesis Planning Using Data‐Driven Learning,” Nature Communications 13, no. 1 (2022): 964, 10.1038/s41467-022-28536-w. PMC8857209 35181654

[29] T. Yu , A. G. Boob , M. J. Volk , X. Liu , H. Cui , and H. Zhao , “Machine Learning‐Enabled Retrobiosynthesis of Molecules,” Nature Catalysis 6, no. 2 (2023): 137–151, 10.1038/s41929-022-00909-w.

[30] W. Finnigan , L. J. Hepworth , S. L. Flitsch , and N. J. Turner , “RetroBioCat as a Computer‐Aided Synthesis Planning Tool for Biocatalytic Reactions and Cascades,” Nature Catalysis 4, no. 2 (2021): 98–104, 10.1038/s41929-020-00556-z. PMC7116764 33604511

[31] K. Sankaranarayanan , E. Heid , C. W. Coley , D. Verma , W. H. Green , and K. F. Jensen , “Similarity Based Enzymatic Retrosynthesis,” Chemical Science 13, no. 20 (2022): 6039–6053, 10.1039/D2SC01588A. 35685792 PMC9132021

[32] Y. Jia , R. Frydrych , Y. I. Sobolev , et al., “Robot‐assisted Mapping of Chemical Reaction Hyperspaces and Networks,” Nature 645, no. 8082 (2025): 922–931, 10.1038/s41586-025-09490-1. 40993255 PMC12460177

[33] B. Prajapati , W.‐S. Wong , R. Frydrych , et al., “Robot‐Assisted Reconstruction and Control of the Pechmann Reaction Network,” Angewandte Chemie International Edition 65, no. 6 (2026): 18394, 10.1002/anie.202518394. 41387232

[34] Y. Jiang , L. Gadina , J. C. Ahumada , et al., “Hyperspace Mapping and Iterative Dimensionality Reduction Reveal Radical Cascade Networks Controlled by Perovskite‐Type Catalysts,” Chem 12 (2026): 102987.

[35] N. Onishchenko , E. S. Larsen , G. Paneru , et al., “Robots and Minimal, Physics‐Informed Features: A Hybrid Framework for Enzyme Catalysis, Zenodo Data Repository,” Version 1 (2025), 10.5281/zenodo.15846762.

[36] S. B. Raj , S. Ramaswamy , and B. V. Plapp , “Yeast Alcohol Dehydrogenase Structure and Catalysis,” Biochemistry 53 (2014): 5791–5803. 25157460 10.1021/bi5006442 PMC4165444

[37] B. V. Plapp and S. Ramaswamy , “Atomic‐Resolution Structures of Horse Liver Alcohol Dehydrogenase with NAD+ and Fluoroalcohols Define Strained Michaelis Complexes,” Biochemistry 51, no. 19 (2012): 4035–4048, 10.1021/bi300378n. 22531044 PMC3352959

[38] S. Calvelage , M. Dörr , M. Höhne , and U. T. Bornscheuer , “A Systematic Analysis of the Substrate Scope of ( S )‐ and ( R )‐Selective Amine Transaminases,” Advanced Synthesis & Catalysis 359, no. 23 (2017): 4235–4243, 10.1002/adsc.201701079.

[39] V. Smer‐Barreto , A. Quintanilla , R. J. R. Elliott , et al., “Discovery of Senolytics Using Machine Learning,” Nature Communications 14, no. 1 (2023): 1–15, 10.1038/s41467-023-39120-1. PMC10257182 37301862

[40] D. Stumpfe , H. Hu , and J. Bajorath , “Advances in Exploring Activity Cliffs,” Journal of Computer‐Aided Molecular Design 34, no. 9 (2020): 929–942, 10.1007/s10822-020-00315-z. 32367387 PMC7367915

[41] D. Van Tilborg , A. Alenicheva , and F. Grisoni , “Exposing the Limitations of Molecular Machine Learning With Activity Cliffs,” Journal of Chemical Information and Modeling 62 (2022): 5938–5951. 36456532 10.1021/acs.jcim.2c01073 PMC9749029

[42] B. J. Bender , S. Gahbauer , A. Luttens , et al., “A Practical Guide to Large‐Scale Docking,” Nature Protocols 16, no. 10 (2021): 4799–4832, 10.1038/s41596-021-00597-z. 34561691 PMC8522653

[43] A. Díaz‐Holguín , M. Saarinen , D. D. Vo , et al., “AlphaFold Accelerated Discovery of Psychotropic Agonists Targeting the Trace Amine‐Associated Receptor 1,” Science Advances 10 (2024): adn1524. 10.1126/sciadv.adn1524 PMC11305387 39110804

[44] A. A. Sadybekov , A. V. Sadybekov , Y. Liu , et al., “Synthon‐Based Ligand Discovery in Virtual Libraries of over 11 Billion Compounds,” Nature 601, no. 7893 (2022): 452–459, 10.1038/s41586-021-04220-9. 34912117 PMC9763054

[45] J. Lyu , S. Wang , T. E. Balius , et al., “Ultra‐Large Library Docking for Discovering New Chemotypes,” Nature 566, no. 7743 (2019): 224–229, 10.1038/s41586-019-0917-9. 30728502 PMC6383769

[46] D. Santos‐Martins , S. Forli , M. J. Ramos , and A. J. Olson , “AutoDock4_Zn_: An Improved AutoDock Force Field for Small‐Molecule Docking to Zinc Metalloproteins,” Journal of Chemical Information and Modeling 54, no. 8 (2014): 2371–2379, 10.1021/ci500209e. 24931227 PMC4144784

[47] A. Scarpino , G. G. Ferenczy , and G. M. Keserü , “Comparative Evaluation of Covalent Docking Tools,” Journal of Chemical Information and Modeling 58, no. 7 (2018): 1441–1458, 10.1021/acs.jcim.8b00228. 29890081

[48] A. T. McNutt , Y. Li , R. Meli , R. Aggarwal , and D. R. Koes , “GNINA 1.3: The Next Increment in Molecular Docking with Deep Learning,” Journal of Cheminformatics 17, no. 1 (2025): 28, 10.1186/s13321-025-00973-x. 40025560 PMC11874439

[49] G. A. Aleku , C. Prause , R. T. Bradshaw‐Allen , et al., “Terminal Alkenes From Acrylic Acid Derivatives via Non‐Oxidative Enzymatic Decarboxylation by Ferulic Acid Decarboxylases,” Chemcatchem 10, no. 17 (2018): 3736–3745, 10.1002/cctc.201800643. 30333895 PMC6175315

[50] H. Huang , C. Pandya , C. Liu , et al., “Panoramic View of a Superfamily of Phosphatases through Substrate Profiling,” Proceedings of the National Academy of Sciences 112 (2015): E1974–E1983. 10.1073/pnas.1423570112 PMC4413258 25848029

[51] M. Martinez‐Martinez , C. Coscolin , G. Santiago , et al., “Determinants and Prediction of Esterase Substrate Promiscuity Patterns,” ACS Chemical Biology 13, no. 1 (2017): 225–234, 10.1021/acschembio.7b00996. 29182315

[52] I. Barrio‐Hernandez , J. Yeo , J. Janes , et al., “Clustering Predicted Structures at the Scale of the Known Protein Universe,” Nature 622, no. 7983 (2023): 637–645, 10.1038/s41586-023-06510-w. 37704730 PMC10584675

[53] S. Szymkuć , A. Wołos , R. Roszak , and B. A. Grzybowski , “Estimation of Multicomponent Reactions′ Yields from Networks of Mechanistic Steps,” Nature Communications 15 (2024): 10286. 10.1038/s41467-024-54550-1 PMC11603315 39604372

[54] B. Mikulak‐Klucznik , P. Gołębiowska , A. A. Bayly , et al., “Computational Planning of the Synthesis of Complex Natural Products,” Nature 588, no. 7836 (2020): 83–88, 10.1038/s41586-020-2855-y. 33049755

[55] T. Klucznik , B. Mikulak‐Klucznik , M. P. McCormack , et al., “Efficient Syntheses of Diverse, Medicinally Relevant Targets Planned by Computer and Executed in the Laboratory,” Chemistry 4, no. 3 (2018): 522–532, 10.1016/j.chempr.2018.02.002.

[56] F. Strieth‐Kalthoff , S. Szymkuć , K. Molga , A. Aspuru‐Guzik , F. Glorius , and B. A. Grzybowski , “Artificial Intelligence for Retrosynthetic Planning Needs Both Data and Expert Knowledge,” Journal of the American Chemical Society 146 (2024): 11005–11017. 10.1021/jacs.4c00338 38598363

[57] M. Moskal , W. Beker , S. Szymkuć , and B. A. Grzybowski , “Scaffold‐Directed Face Selectivity Machine‐Learned from Vectors of Non‐Covalent Interactions,” Angewandte Chemie International Edition 60, no. 28 (2021): 15230–15235, 10.1002/anie.202101986. 33876554

[58] W. Beker , E. P. Gajewska , T. Badowski , and B. A. Grzybowski , “Prediction of Major Regio‐, Site‐, and Diastereoisomers in Diels–Alder Reactions by Using Machine‐Learning: The Importance of Physically Meaningful Descriptors,” Angewandte Chemie International Edition 58, no. 14 (2019): 4515–4519, 10.1002/anie.201806920. 30398688

[59] J. Abramson , J. Adler , J. Dunger , et al., “Accurate Structure Prediction of Biomolecular Interactions with AlphaFold 3,” Nature 630, no. 8016 (2024): 493–500, 10.1038/s41586-024-07487-w. 38718835 PMC11168924

[60] S. Passaro , G. Corso , J. Wohlwend , et al., “Boltz‐2: Binding Affinity Prediction,” bioRxiv (2025): 659707, 10.1101/2025.06.14.659707.

