# Supplementary material for: Robots and Minimal, Physics‐Informed Features: A Hybrid Framework for Enzyme Catalysis
Source: Adv Sci (Weinh). 2026 Sep 11:e77474. Online ahead of print. doi: 10.1002/advs.77474 (PMC13564181; doi:10.1002/advs.77474)
Supplement: Supplementary file 1 [file ADVS-9999-e77474-s002.pdf]

Supporting Information for Manuscript titled:

Robots and Minimal, Physics-Informed Features: A Hybrid Framework for Enzyme Catalysis

Natalia Onishchenko[1†], Eric S. Larsen[1†], Govind Paneru[1,2†], Kisung Lee[1†], Diana Kolygina[1], Yankai Jia[1], Elizabeth Maria Clarissa[1,3], Wai-Shing Wong[1], and Bartosz A. Grzybowski[1,4]*

[1] Center for Algorithmic and Robotized Synthesis, Institute for Basic Science (IBS), Ulsan, Republic of Korea

[2] Department of Physics, Ulsan National Institute of Science and Technology, Ulsan, Republic of Korea

[3] Department of Biomedical Engineering, Ulsan National Institute of Science and Technology, Ulsan, Republic of Korea

[4] Department of Chemistry, Ulsan National Institute of Science and Technology, Ulsan, Republic of Korea

[†] These authors contributed equally

*Correspondence to: nanogrzybowski@gmail.com

EXPERIMENTAL DETAILS

1. Materials. Alcohol dehydrogenase from *Saccharomyces cerevisiae* (yADH, cat. no. A3263), horse liver alcohol dehydrogenase (hlADH, cat. no. 55689), ω-transaminase from *Aspergillus terreus* (TA-At, cat. no. 40972) and *Aspergillus fumigatus* (TA-Af, cat. no. 77087), were purchased from Sigma-Aldrich (US). Substrates were purchased from one of the vendors: Sigma-Aldrich, Ambeed, BLD Pharma, 10X Chem, or TCI. Corning® Costar® Ultra-Low Attachment Multiple Well Plates, 96 well (CLS3474) were used for the assays. Hamilton™ CO-RE™ II Conductive Filter Tips, non-sterile, were used for pipetting. For all the solutions and assays, deionized water (Milli-Q Integral 15 water purification system) was used. Tris buffer (TB) was prepared by dilution of 1 M stock, pH 8.0 (Biosesang, South Korea). HEPES buffer (HB) contained 11.915 g HEPES (Sigma-Aldrich) per 1 L water, pH adjusted to 8.0. HPLC-grade solvents were purchased from Sigma and used without further purification.

2. Enzyme activity assays. Assays were selected so that they could be easily performed in the 96-well format, without interference of substrates and/or products of the reaction with the readout. For ADH's, this meant a straightforward use of the NADH product absorbance (Supplementary **Figure S12**). For transaminases, however, the most widely used assay relies on 1-phenylethylamine as the second substrate and the absorbance of the acetophenone product in the region of 240–280 nm—this method requires UV-transparent plates and does not allow for assaying aromatic compounds as substrates since they normally absorb in the region of interest. Hence, we employed a fluorescence-based assay[1] utilizing 1-(6-methoxynaphth-2-yl)ethylamine as the second substrate (Supplementary **Figure S13**).

To account for possible edge effects and errors due to evaporation, sample placement over the plate was randomized.

2.1. Alcohol dehydrogenase (ADH) assay. Concentrated substrate solutions (1 M) were prepared in DMSO and then diluted ten-fold with TB. Reaction mixture contained 31.25 mM substrate, 62.5 µg/mL yADH or 625 µg/mL hlADH, and 1 mM NAD (Sigma-Aldrich, N0632) in TB-DMSO buffer (10% DMSO in 10 mM Tris buffer, pH 8.0). To push the reaction forward, high excess of alcohol was used. The solutions were pipetted using an in-house-built Robowski platform in groups of 12 substrates, each in triplicate. Control wells contained TB-DMSO instead of the substrate solution. Each run contained wells with the reference substrate (ethanol) as a positive control. Immediately after the end of pipetting, the plate was transferred to the M200 plate reader (TECAN, Austria) preequilibrated at 25°C. Absorbance of NADH generated in the course of the reaction was read at 340 nm for 1 h. Equilibrium yields were calculated with respect to the starting NAD concentration using NADH calibration curve.

2.2. ω-Transaminase (TA) assay. Concentrated substrate (amine acceptor) solutions (1.2 M) were prepared in DMSO and then diluted 120-fold with HEPES buffer. Reaction mixture contained 1.25 mM amine acceptor, 20 µg/mL oTA-At or 40 µg/mL oTA-Af, 5 µM pyridoxal phosphate (PLP), and 0.5 mM (R)-1-(6-methoxynaphthalen-2-yl)ethanamine (MNEA; BLD Pharm, BD77489; amine donor) in HEPES buffer, 50 mM, pH 8.0 (HB); total volume was 250 µL. The solutions were pipetted using an in-house-built Robowski platform in groups of 12 substrates, each in triplicate. Each run contained wells with the reference substrate (pyruvate) as a positive control. Immediately after the end of pipetting, the plate was transferred to the M200 plate reader (TECAN, Austria) preequilibrated at 28°C. Fluorescence intensity (λex 330 nm, λem 460 nm) of the ketone generated in the course of the reaction was read for 50 min. Equilibrium yields were calculated with respect to starting MNEA concentration using the 2-acetyl-6-methoxynaphthalene (Ambeed) calibration curve.

3. Docking substrates to enzymes. 3D SDF files of the substrates were downloaded from the PubChem database using the PubChemPy module in Python. Each substrate's coordinates were converted to PDB

format using OpenBabel[2], followed by conversion to PDBQT format using AutoDock tools (ADT v1.5.7)[3], specifically employing the prepare_ligand4.py script with its default settings on a Linux machine to merge non-polar hydrogens, add Gasteiger–Marsili charges, and assign atom types.

Structure of all enzymes were obtained from the Protein Data Bank (PDB) and pre-processed following standard AutoDock procedures. For yeast alcohol dehydrogenase (yADH, PBD ID 7KCB) and horse liver alcohol dehydrogenase (hlADH, PDB ID 2OHX) enzymes, which contain a catalytic Zn^{2+} ion and the oxidized nicotinamide cofactor NAD^+ (component NAD) at their active sites, hydrogens were added using Reduce software[4] with the NOFLIP option. The Zn^{2+} ion and cofactor were retained in their native crystallographic positions throughout docking; the co-crystallized small molecules occupying the substrate site (trifluoroethanol in 7KCB, DMSO in 2OHX) were removed beforehand. Docked poses were evaluated against the reference substrate (ethanol) for consistency with a productive hydride-transfer geometry – orientation of the hydride-donating carbon toward C4 of the nicotinamide ring, with the reactive hydroxyl directed toward the catalytic Zn^{2+}. For substrates with multiple hydroxyl groups, the hydroxyl nearest the catalytic Zn^{2+} was used to define the reactive head group for the RMSD and angular-deviation measurements. The remaining two enzymes, namely ω-transaminases from *A. terreus* (TA-At, PDB ID 4CE5) and *A. fumigatus* (TA-Af, 4CHI), which lack metal centers, were preprocessed using the PDB2PQR software[5]. The ADT tool prepare_receptor4.py script, with default settings, was used to remove water molecules, add Gasteiger–Marsili charges, merge non-polar hydrogens, and assign atom types. Transaminases were prepared by converting the cofactor pyridoxal 5'-phosphate (PLP) to its reduced form, pyridoxamine 5'-phosphate (PMP), through cleavage of the Schiff base linkage between PLP and the enzyme, followed by reduction of the resulting imine to a protonated amine ($–NH_3^+$), introducing a +1 charge (see Supplementary Figure S15) using PyMOL software[6,7].We emphasize that docking carbonyl substrates into this fixed PMP-bound active site is a practical surrogate for evaluating substrate orientation and electronic complementarity at the reaction center; it is not a mechanistic model of transamination. The covalent Schiff-base (aldimine) intermediates, proton-transfer steps, and dynamic PLP/PMP cofactor interconversion that constitute the catalytic cycle are not represented. As for the other enzyme families, the docking model serves only to assess whether a candidate substrate's reactive headgroup can attain a productive, reference-like orientation, which we treat as a necessary precondition for rather than a guarantee of catalysis. Docking substrates to yADH and hlADH was performed using AutoDock4Zn[8], while docking to the other two enzymes utilized AutoDock 4.2.6. Docking simulations were executed using a custom Python script in parallel across 24 CPU cores with the genetic algorithm (GA) search settings at default values, except the number of poses generated (ga_runs) was set to 100. The grid box size was set to 30 × 30 × 30 with grid spacing of 0.375 Å.

For the external dataset, data was extracted from a paper on ferulic acid decarboxylases[9]. The ligand-bound crystal structure of *Aspergillus niger* FDC (PDB ID4ZAB) was used as the receptor for docking. Chain A was isolated, crystallographic waters and all cofactors except 4LU were removed, and hydrogens were added using the Reduce software with the -NOFLIP option. This procedure preserved the deposited heavy-atom residue identities while generating a hydrogenated, single-conformer receptor model for docking. Ligand structures were obtained in SDF format and converted to the carboxylate anion form (COO^-) prior to PDBQT generation. The docking was done by choosing 40 × 40 × 40 grid points with a spacing of 0.375 Å. Grid center was at default coordinates (19.825, 8.899, 20.91).

For the second external dataset, ligand SMILES were extracted from[10] and verified. SMILES of the stereochemically ambiguous molecules were removed from the dataset. Six- and seven-carbon sugars were considered to predominantly exist as pyranose ring forms, with furanose forms favored when the C5 atom was phosphorylated or otherwise functionalized. Ligand structures were downloaded from the

PubChem database in 3D SDF format. Only monophosphate-containing substrates were selected for analysis. All ligands were prepared in their dianionic form, corresponding to a total charge of –2.

The initial dataset has 218 phosphatases screened against 165 substrates. We chose one of the enzymes with more than 30% positives according to the authors' cutoff. The crystal structure of the putative *Escherichia coli* phosphatase (PDB ID 2B0C) was prepared in PyMOL. All co-crystallized ligands were removed prior to docking. Crystallographic water molecules were removed except for those within 3.5 Å of any atom of the bound 1-*O*-phosphono-alpha-D-glucopyranose (G1P) ligand in the crystal structure, thereby retaining the ordered "structural" waters most likely to stabilize the experimental binding pose while excluding bulk solvent waters that are unlikely to be conserved.

Docking parameters were validated by redocking the native G1P ligand in dianionic form into the active site. The grid center was defined as the centroid of G1P (21.604, 4.019, 52.643), and the search space was set to 40 × 40 × 40 grid points with a spacing of 0.375 Å. Under these conditions, the docked G1P pose reproduced the crystallographic binding mode with an RMSD of 0.8 Å relative to the crystal pose.

Because direct SDF-to-PDBQT conversion did not reliably preserve the charge state of several dianionic phosphate-containing ligands, a two-step ligand preparation procedure was used. Ligands were first converted from SDF to MOL2 format using Open Babel with Gasteiger partial charges. The summed atomic partial charges of each MOL2 file were then checked to confirm consistency with the expected total ligand charge. The MOL2 files were subsequently converted to PDBQT format using MGLTools 1.5.7 while preserving the input partial charges rather than recalculating them. Finally, the total charge was rechecked after PDBQT generation to confirm charge preservation within a tolerance of 0.01 charge units.

The third literature dataset was extracted from.[11] Ligand structures were downloaded from the PubChem database in 3D SDF format. The crystal structure of *Candida antarctica* lipase B (CALB; PDB ID: 1LBS) was retrieved from the Protein Data Bank. In this structure, the catalytic serine is covalently trapped by the phosphonate transition-state analogue HEE (ethyl hydrogen hexylphosphonate). The asymmetric unit contains six crystallographically independent chains (A–F); only chain A was retained for docking. Using PyMOL v3.0.0, all non-protein components, including the covalently bound HEE adduct, ordered water molecules, ions, and buffer-derived heteroatoms, were removed, leaving only the polymeric protein atoms of chain A.

Because the HEE phosphorus is covalently bonded to the Ser105 Oγ atom in the deposited structure (P–Oγ = 1.56 Å), removal of the inhibitor restored the catalytic Ser105 residue with a free Oγ nucleophile. Therefore, no additional repair of the catalytic residue was required.

The docking search space was defined as a 70 × 70 × 70-point affinity grid with 0.375 Å spacing, corresponding to approximately 26 Å per edge. The grid was centered on the centroid of the HEE ligand coordinates (x = 55.69, y = 3.00, z = 34.63 Å), which coincides with the catalytic pocket.

Rotatable bonds were perceived automatically. Amide and guanidinium bonds were kept rigid, whereas all remaining acyclic single bonds were treated as active torsions. Substrates whose active-torsion count exceeded the AutoDock4 limit of 32 (compounds 6 and 95), together with cationic substrates excluded by design (compounds 22 and 23), were omitted from the docking analysis. This left 92 ester substrates for docking and feature extraction.

For the esterase campaign, AutoDock was run with 2.5×10^7 energy evaluations per run. All other enzyme-docking campaigns used 2.5×10^6 energy evaluations per run. The population size and the number of Lamarckian genetic algorithm runs were kept identical across all enzyme families.

4. Preparation of input features for supervised learning. Docking results were analyzed by considering both predicted binding energy and pose consistency across docking runs. For each ligand, the representative pose from each cluster was examined, and selection of the final docked pose was performed using a decision-tree procedure designed to balance low binding energy with pose consistency. The representative of the largest cluster was selected if its binding energy was within 0.5 kcal/mol of the minimum-energy pose and the cluster contained at least 10% of all docking runs. The representative of the largest cluster was also selected when that cluster accounted for at least 50% of all runs and its binding energy was within 1.0 kcal/mol of the minimum-energy pose. If neither of these conditions was satisfied, the minimum-energy pose was selected. Thus, the final pose corresponded either to the lowest-energy conformation or, when sufficiently similar in energy and strongly supported by clustering, to the most reproducible binding mode.

These selected poses were saved in the SDF format to generate features for supervised learning from geometric descriptors of the docked substrates relative to a reference substrate. Where available, native substrate was used as a reference one, e.g., ethanol (ADH-2) for ADHs. In case of transaminases, pyruvate (TA-1) served a reference substrate for ketones, while the reasonably well performing picolinaldehyde (TA-14) was used as a reference for aldehydes. For substrates with multiple headgroups, the headgroup nearest the Zn atom was selected for ADHs and the headgroup nearest to the PMP cofactor's N01 atom was selected for TAs for computing the features. Initial 3D SDF files were used as starting conformers for quantum mechanical (QM) optimizations, except for the substrates with multiple headgroups where docked conformations were utilized. All quantum-chemical descriptors (HOMO and LUMO energies and natural bond orbital (NBO) partial charges) were computed for the neutral forms of the substrates in the gas phase at the B3LYP/def2-TZVP level using Gaussian16 for all substrates, applied consistently across all enzyme families, including the external FDC and phosphatase datasets. Neutral forms were used because the gas-phase protocol does not reliably describe ionized species (in particular anionic carboxylates and phosphates), and because a rigorous treatment of assay-pH protonation states would require implicit-solvent calculations that proved computationally prohibitive for the phosphatase substrates. Because descriptors enter the model as differences relative to a reference substrate within each family, substituent-driven trends are expected to be largely preserved across protonation states; the consistent neutral-form treatment is nonetheless noted as a limitation.

Within the framework of our docking model, one can imagine different sets of features. In the beginning of the project, we considered a broad feature range including: RMSD of the headgroup atoms, angular deviation for the reactive bond, and binding energy or differences thereof between the substrate and the reference; partial charge, (Eucledean) distance between substrate and reference for oxygen and carbon atoms of the reactive bond; HOMO and LUMO energies and the gap between them, differences between HOMO and LUMO between the substrate and the reference from QM calculations; binding energy, partial charges and HOMO/LUMO energies of the products. We also considered substrate volume, total polar surface area, and number of hydrogen bonds calculated using RDKit. At early stages of model development, RDKit descriptors, as well as absolute geometry and electronics features, were discarded because they did not improve classification (compared to using only docking- and QM-derived features). Correlation matrix for the resulting set of eight relative (candidate substrate vs. reference) features is shown in **Figure S17**. To choose between the correlating features, they were subjected to feature ablation and Shapley-based feature importance was derived from the ablation experiments on exhaustive set of combinations of training and testing datasets (e.g., ADH + TA → FDC, Pase; ADH + FDC → TA, Pase, and so on; see **section 5** below for details).

(Z)-2-Fluoro-3-phenylacrylic acid (FDC-22) was used as a reference substrate. For each docked ligand, the carboxyl/carboxylate carbon and the adjacent alpha-, beta-, and gamma-carbons were identified from the molecular topology, and RMSD between the reference substrate and each other substrate was calculated using the coordinates of these four atoms. Angular deviation was defined as the angle between the corresponding alpha-C-to-beta-C vectors in the reference and comparison substrates. For ligands with multiple possible matching groups, the substrate geometry used for analysis was the one with the shortest distance between the carboxyl carbon and the C4A atom of 4LU in the receptor. In addition, the NBO charge of the beta-C and HOMO, LUMO energies were computed for the neutral substrates using Gaussian16 at the B3LYP/def2-TZVP level of theory.

D-Glucose-1-phosphate (Pase-26) was used as a reference substrate. Docking pose similarity was quantified by calculating the RMSD between the docked reference substrate and each other docked substrate using the phosphorus P atom and the bridging phosphate oxygen O atom connecting the phosphate group to the substrate carbon C backbone. Angular deviation was defined as the angle between the corresponding O–P vectors of the reference and comparison substrates. Energetic variation was represented as the difference between the docking binding energy of the reference substrate and that of each other substrate. Electronic descriptors were obtained from quantum chemical calculations on the neutral forms of the substrates. Specifically, NBO partial charges of the O atoms and HOMO and LUMO energies were calculated using Gaussian16 at the B3LYP/def2-TZVP level of theory.

For esterase, high yielding ethyl hexanoate (EST-14) was used as a reference substrate. For each docked substrate, the reactive ester group was identified using the SMARTS pattern [CX3]([#6])(=[OX1])[OX2][#6]. The five matched atoms were, in order: (1) the carbonyl carbon, (2) the acyl-side carbon, (3) the carbonyl oxygen, (4) the ester oxygen, and (5) the O-alkyl carbon. When a substrate contained more than one ester group, the group whose carbonyl carbon was closest to the Ser105 Oγ nucleophile was selected for feature calculation. Pose similarity was quantified by comparing the reactive ester group of each docked substrate with that of the reference substrate. The pose RMSD was calculated over the four-atom ester core consisting of the carbonyl carbon, acyl-side carbon, carbonyl oxygen, and ester oxygen. The fifth atom, the O-alkyl carbon, was excluded from the RMSD calculation. Because all substrates were docked into the same rigid receptor frame, RMSD values were calculated directly from the docked coordinates without additional structural superposition. This descriptor therefore measures how far each substrate's reactive ester headgroup is displaced from that of the reference substrate within the CALB active site. Angular Deviation 1 corresponds to the C=O vector. Electronic descriptors were obtained from quantum chemical calculations on the neutral forms of the substrates. Specifically, NBO partial charges of the O atoms and HOMO and LUMO energies were calculated using Gaussian16 at the B3LYP/def2-TZVP level of theory.

5. ML model description. Compounds were classified based on their yield, where a yield >1% was defined as active, and a yield ≤1% was defined as inactive, except for phosphatase literature data, for which active and inactive substrates were those with absorbance >0.2 and absorbance ≤ 0.2, respectively. For esterase, enzyme specific activity was reported for each substrate; substrates were classified as active if the value was more than 0.

Binary classification models were implemented in Python using scikit-learn and, where applicable, XGBoost[12] packages. Four classifiers were evaluated within a common reproducible framework: logistic regression (LR), decision tree (DT), random forest (RF)[13], and XGBoost (XGB). Reaction yield was binarized using predefined cutoffs applied separately to the training and external holdout datasets. Features were standardized (zero mean, unit variance) prior to model training. Class imbalance

was addressed using model-appropriate weighting schemes (balanced class weights for tree-based models, sample weights for logistic regression, and scale_pos_weight for XGBoost).

Optuna[14] with the default TPE (Tree-structured Parzen Estimator) sampler was used for hyperparameter optimization (50–100 trials) of the models. The optimization objective was to maximize balanced accuracy on the validation dataset (VAL BA). The model-specific hyperparameter grids used for LR, DT, RF, and XGB are summarized in Table S1.

Five enzyme-class datasets were used: ADH, TA, FDC, Pase, and esterase to select the best model and feature set. They were split into two training (TRAIN), one validation (VAL), and two test (TEST) family-wise. An exception was a few cases when we used only one enzyme family to TRAIN and VAL (e.g., Figure 4), 5-fold CV was performed within the family for hyperparameter optimization, and the best hyperparameter was used to retrain the model on all substrates of this enzyme family.

Given 8 features (RMSD, ANGLE, GAP, BE, HOMO, LUMO, CHARGE1, CHARGE2) and 4 architectures (LR, DT, RF, and XGB), we first selected the optimal feature set and architecture. For this purpose, we trained models on exhaustive combinations of 2 TRAIN / 1 VAL enzyme families. It resulted in a total of 30 TRAIN/VAL combinations, where each enzyme family had corresponding 6 unique combinations of TRAIN enzyme families (e.g., ADH + TA → FDC; ADH + Pase → FDC; Pase + TA → FDC; ADH + Esterase → FDC; TA + Esterase → FDC; Pase + Esterase → FDC). Prediction for each substrate was obtained via soft-vote (where every substrate gets an average prediction score over 6 models), resulting in a performance metric for each enzyme family used as a VAL set. The metric used for ranking was 5 VAL averaged BA.

Given the metric, the feature set and architecture were selected in two steps. First, the best performing features were found. The selection was based on sweeping through combinations of features (combinations of 3–8 features, 219 in total), and averaging VAL BA over 4 architectures. It resulted in 219 metrics. Feature set of RMSD, ANGLE, GAP was ranked the highest, as shown in the model performance ranking across top 10 feature combinations (**Figure S8a**). Second, the best performing architecture was found for the selected feature set, by comparing VAL BA of each architecture. The model was chosen as RF, as it is only marginally different from XGBoost (see **Figure S9**), with a significantly less computational cost. **Figure S9a–e** shows CV metrics for the selected feature set over different models.

The selected model (RF architecture and 3 features) was used to obtain TEST set predictions as described in the main text. For each enzyme family as TEST, we have 12 TRAIN/VAL combinations, and for each substrate a prediction score was aggregated similarly over 12 models (i.e., soft-vote; see **Figure S10**). The split mimics the situation when we know 3 enzyme families and use their exhaustive combination to extrapolate to 2 enzyme families that were never seen either in TRAIN or VAL. Splitting the data in 2/1 family and shuffling combination would allow a richer extrapolative signal, as VAL is on extrapolation.

Model performance was evaluated on held-out test sets using a fixed classification threshold of 0.5. Performance metrics included accuracy, balanced accuracy, F1 score, and ROC-AUC, AUCPR, and normalized MCC (nMCC = (1+MCC)/2). For reproducibility, random seeds were fixed (seed = 42) for all steps and cross-validation splits. All experiments were implemented in Python 3.11 using scikit-learn (v1.4.0), XGBoost (≥2.0.0), and Optuna (≥3.5.0).

6. Testing other models. The ProSmith model was accessed via the web interface at deepmolecules.org. VIPER code and parameter setting files were downloaded from git repository and run in Google Colab

(https://colab.research.google.com/drive/1vrTYn5MSS60HcuIs1nRoJpdvR5zITgkL?usp=drive_link). EZSpecificity model was kindly tested with provided data by Prof. Huimin Zhao and Dr. Tanner James Dean. Since a number of these models yield ratings of substrates, downloaded output files were used to calculate AUROC values.

Data and code availability. Structures of all substrates and their yields are listed in the Supporting Information below. Raw data, code to dispense the solutions, collect and analyze the measurement data, dock the substrates, extract features and train models, are deposited at Zenodo repository https://doi.org/10.5281/zenodo.15846763.

SI References

[1] T. Scheidt, H. Land, M. Anderson, Y. Chen, P. Berglund, D. Yi, W. D. Fessner, "Fluorescence-Based Kinetic Assay for High-Throughput Discovery and Engineering of Stereoselective ω-Transaminases" *Adv. Synth. Catal.* **2015**, 357, 1721–1731.

[2] N. M. O'Boyle, M. Banck, C. A. James, C. Morley, T. Vandermeersch, G. R. Hutchison, "Open Babel: An Open Chemical Toolbox" *J. Cheminf.* **2011**, *3*, 33–47.

[3] R. Huey, G. M. Morris, *Using AutoDock 4 with AutoDockTools : A tutorial*, The Scripps Research Institute, La Jolla, California, **2008**, 1–56.

[4] J. M. Word, S. C. Lovell, J. S. Richardson, D. C. Richardson, "Asparagine and Glutamine: Using Hydrogen Atom Contacts in the Choice of Side-Chain Amide Orientation" *J. Mol. Biol.* **1999**, *285*, 1735–1747.

[5] E. Jurrus, D. Engel, K. Star, K. Monson, J. Brandi, L. E. Felberg, D. H. Brookes, L. Wilson, J. Chen, K. Liles, M. Chun, P. Li, D. W. Gohara, T. Dolinsky, R. Konecny, D. R. Koes, J. E. Nielsen, T. Head-Gordon, W. Geng, R. Krasny, G. W.Wei, M. J. Holst, J. A. McCammon, N. A. Baker, "Improvements to the APBS Biomolecular Solvation Software Suite" *Protein Sci.* **2018**, *27*, 112–128.

[6] Q. Dherbassy, R. J. Mayer, K. B. Muchowska, J. Moran, "Metal-Pyridoxal Cooperativity in Nonenzymatic Transamination" *J. Am. Chem. Soc.* **2023**, *145*, 13357–13370.

[7] The PyMOL Molecular Graphics System, Version 2.0, Schrodinger, LLC: New York, NY, **2015**.

[8] D. Santos-Martins, S. Forli, M. J. Ramos, A. J. Olson, "AutoDock4Zn: An Improved AutoDock Force Field for Small-Molecule Docking to Zinc Metalloproteins" *J. Chem. Inf. Model.* **2014**, *54*, 2371–2379.

[9] G. A. Aleku, C. Prause, R. T. Bradshaw-Allen, K. Plasch, S. M. Glueck, S. S. Bailey, K. A. P. Payne, D. A. Parker, K. Faber, D. Leys, "Terminal Alkenes from Acrylic Acid Derivatives via Non-Oxidative Enzymatic Decarboxylation by Ferulic Acid Decarboxylases" *ChemCatChem* **2018**, *10*, 3736–3745.

[10] H. Huang, Ch. Pandya, Ch. Liu, N. F. Al-Obaidi, M. Wang, L. Zheng, S. T. Keating, M. Aono, J. D. Love, B. Evans, R. D. Seidel, B. S. Hillerich, S. J. Garforth, S. C. Almo, P. S. Mariano, D. Dunaway-Mariano, K. N. Allen, J. D. Farelli, "Panoramic View of a Superfamily of Phosphatases through Substrate Profiling" *Proc. Natl. Acad. Sci.*, **2015**, *112*, E1974–E1983.

[11] M. Martinez-Martinez, C. Coscolin, G. Santiago, J. Crow, P.J. Stogios, R. Bargiela, C. Gertler, J. Navarro-Fernandez, A. Bollinger, S.Thies, C. Mendez-Garcia, A. Popovic, G. Brown, T.N. Chernikova, A. Garcia-Moyano, G.E. Bjerga, P. Perez-Garcia, T. Hai, M.V. Del Pozo, R. Stokke, I.H. Steen, H. Cui, X. Xu, B.P. Nocek, M. Alcaide, M. Distase, V. Mesa, A.I. Pelaez, J. sanchez, P.C.F. Buchholz, J. Pleiss,

A. Fernandez-Guerra, F.O. Glockner, O.V. Golyshina, M.M. Yakimov, A.savchenko, K.-E. Jaeger, A.F. Yakunin, W.R. Streit, P.N. Golyshin, C. Guallar, M. Ferrer, the INMARE Consortium, “Determinants and Prediction of Esterase Substrate Promiscuity Patterns” *ACS Chem. Biol*. **2017**, *13*, 225–234.

[12] XGBoost Documentation, can be found under https://xgboost.readthedocs.io/en/stable/install.html, **2022** (accessed September 2, 2025).

[13] L. Breiman, “Random Forests” *Machine Learning* **2001**, *45*, 5–32.

[14] T. Akiba, Sh. Sano, T. Yanase, T. Ohta, M. Koyama, “Optuna: A Next_Generation Hyperparameter Optimization Framework” **2019** *KDD ’19: Proceedings of the 25th ACM SIGKDD International Conference on Knowledge Discovery and Data Mining*, 2623–2631.

SUPPORTING INFORMATION

Movie S1: Automated robotic platform for reaction preparation and characterization. 00:00 – 01:50, automated pipetting of enzymatic reaction components into a 96-well plate. 01:51 – end, transfer of a well plate between the pipetting setup and the plate reader by a robotic arm for measurement. Pipetting planning, robotic arm trajectory control, setup synchronization and data acquisition are coordinated by a custom-built Python program deposited Zenodo (https://doi.org/10.5281/zenodo.15846763).

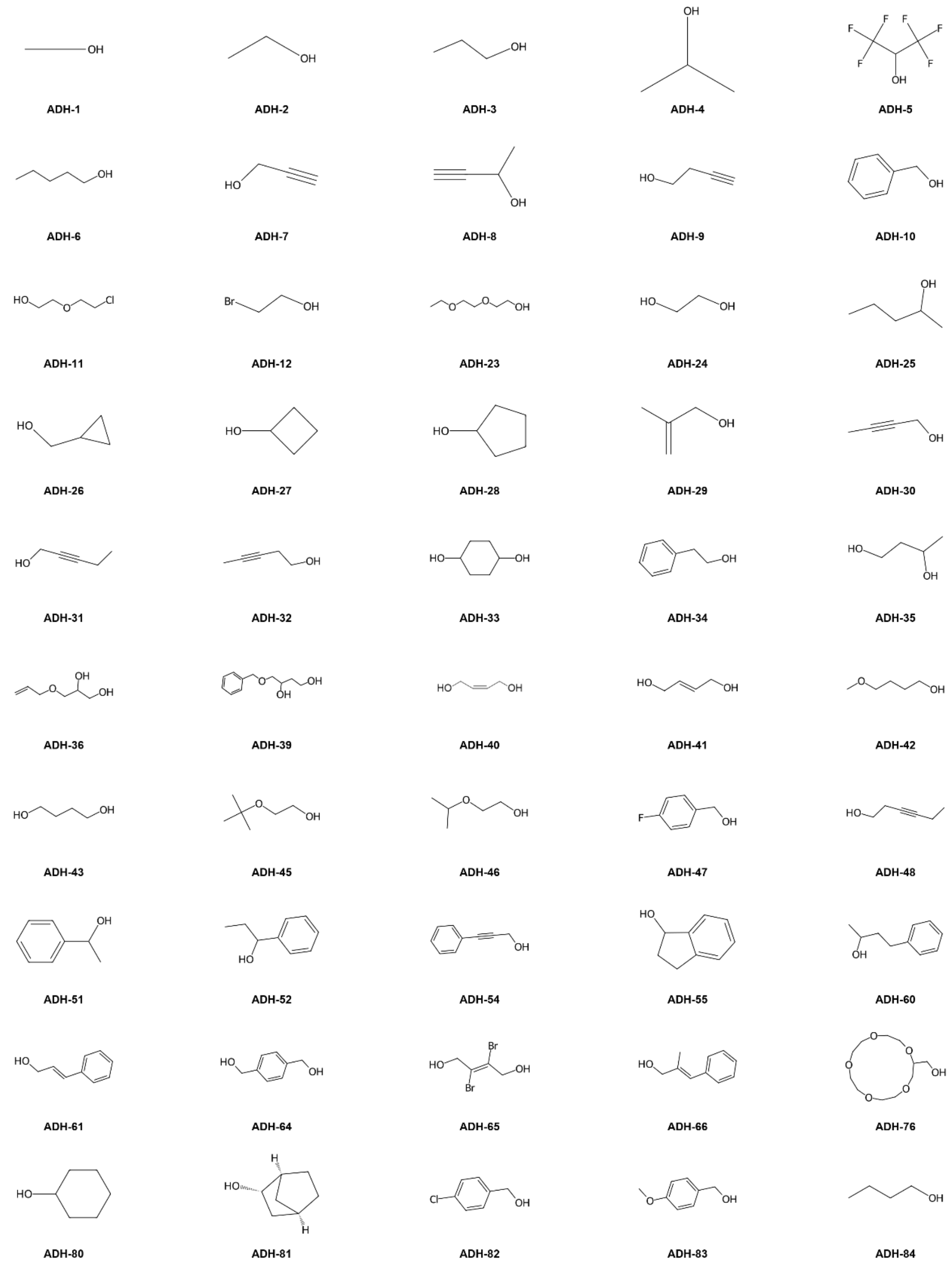

Figure S1: Substrates used for reaction screening with alcohol dehydrogenase enzymes.

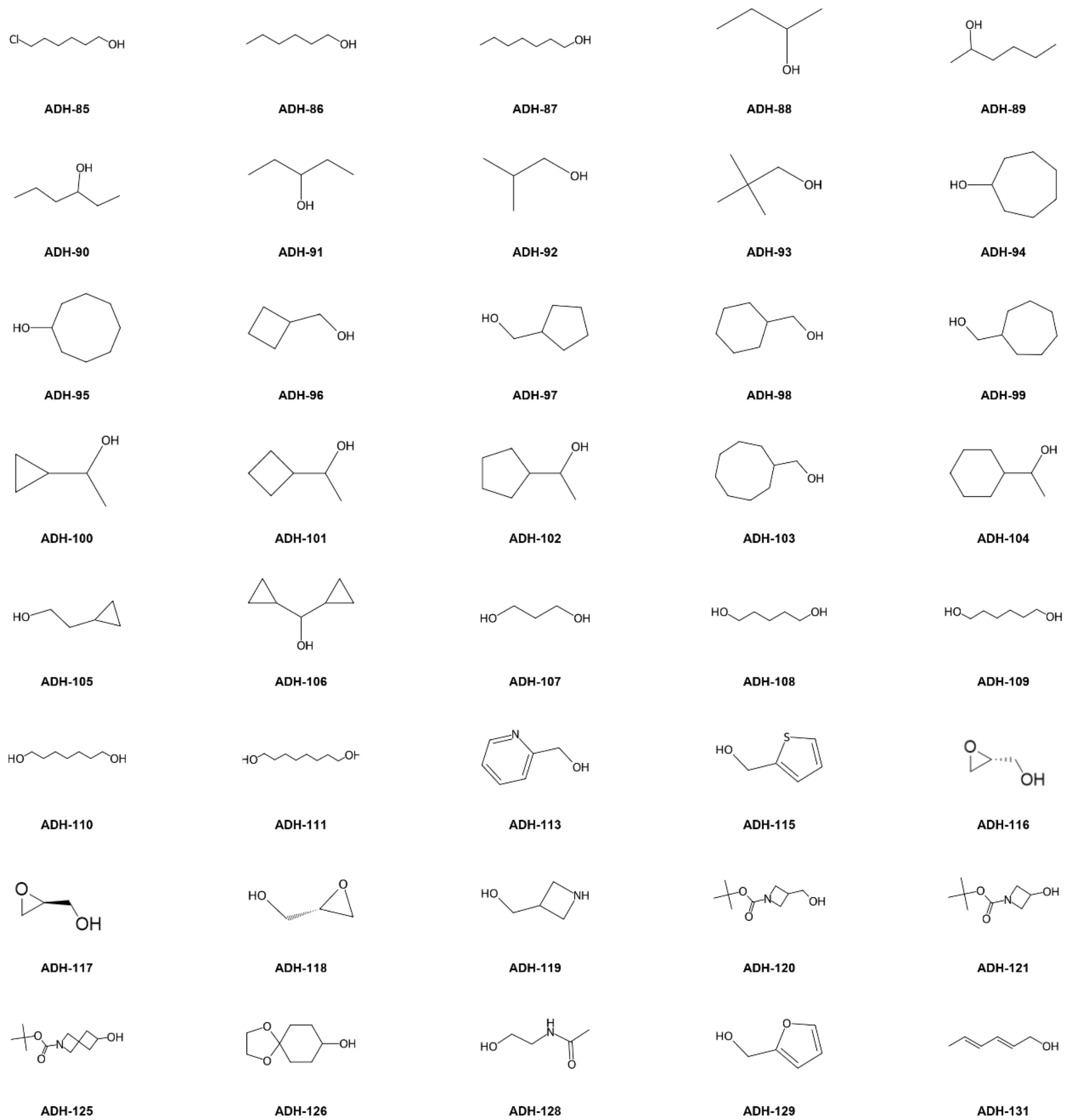

Figure S1 (contd.): Substrates used for reaction screening with alcohol dehydrogenase enzymes.

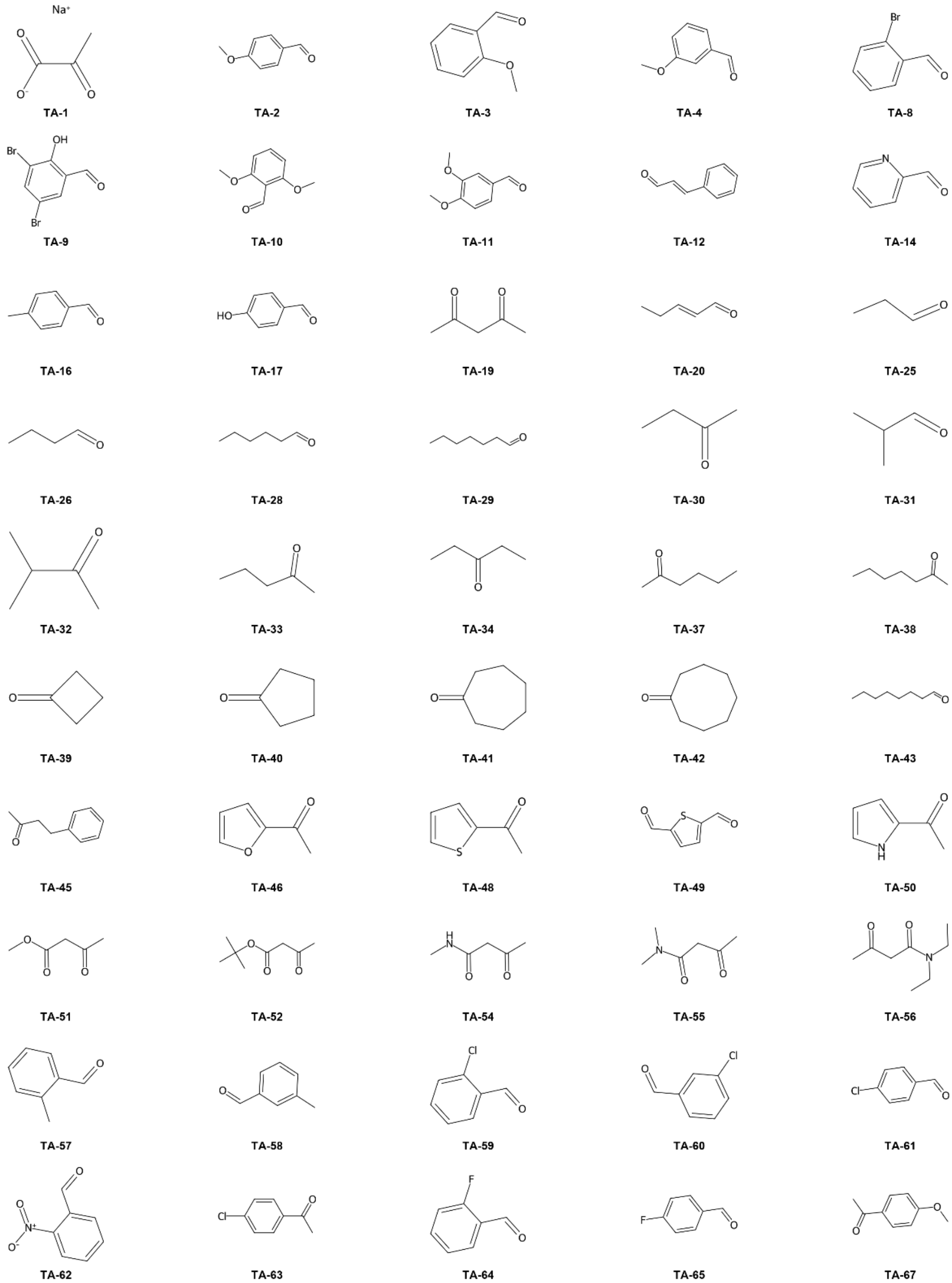

Figure S2: Substrates used for screening with transaminase enzymes.

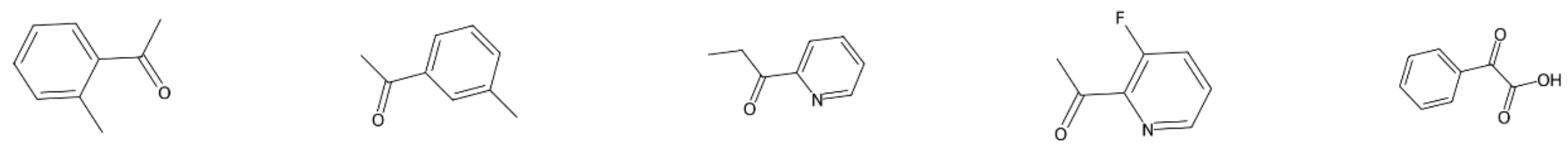

TA-69 TA-70 TA-74 TA-75 TA-79

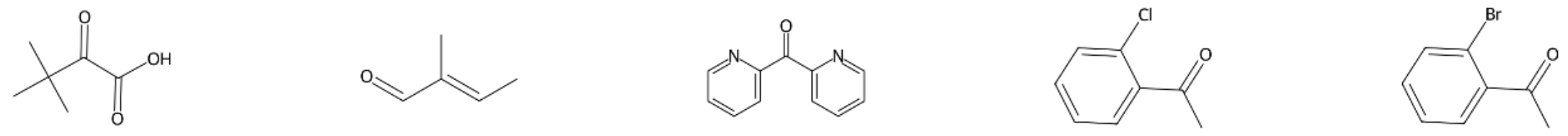

TA-81 TA-82 TA-85 TA-87 TA-88

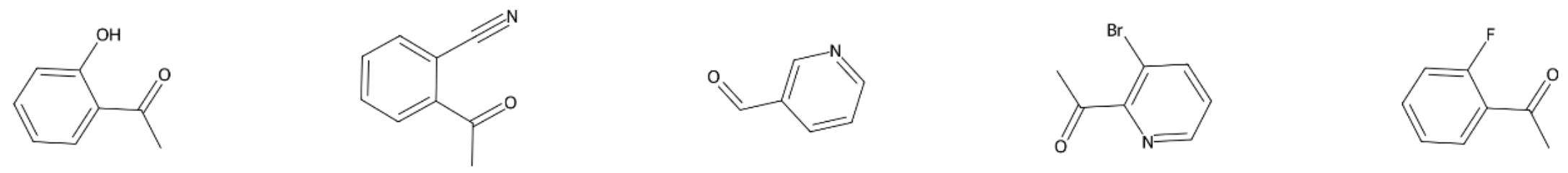

TA-89 TA-90 TA-91 TA-92 TA-93

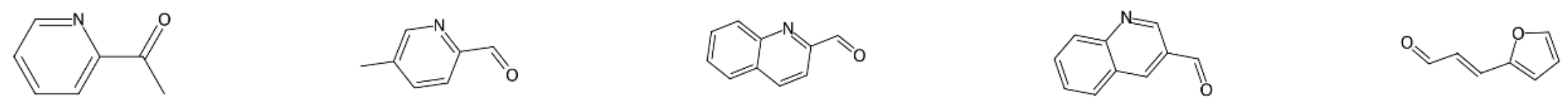

TA-94 TA-95 TA-96 TA-98 TA-99

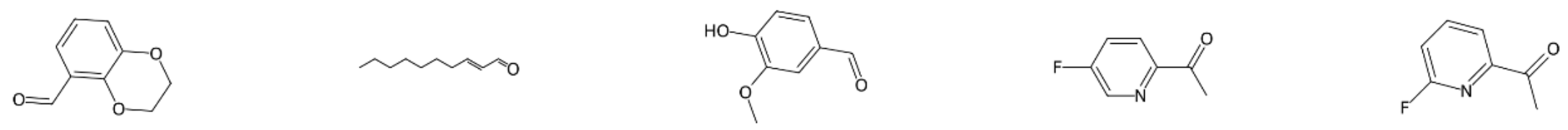

TA-100 TA-101 TA-102 TA-103 TA-104

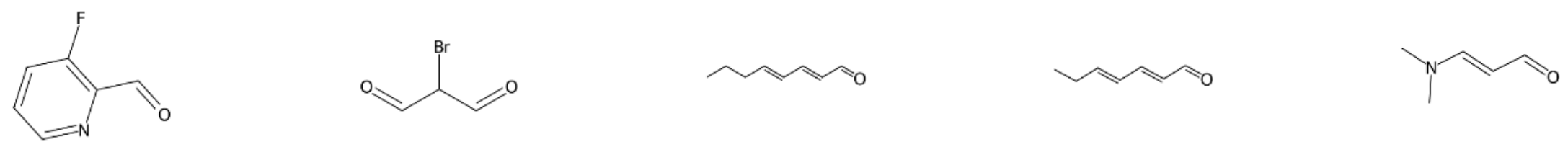

TA-105 TA-106 TA-107 TA-108 TA-109

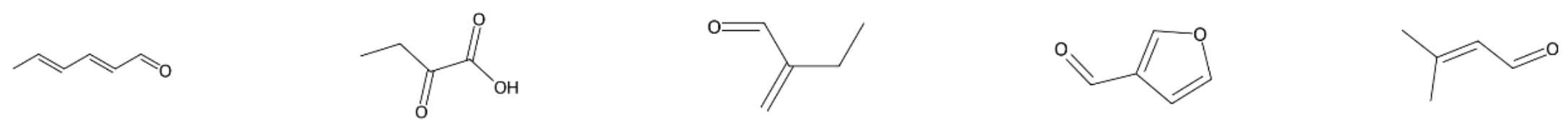

TA-110 TA-111 TA-112 TA-113 TA-114

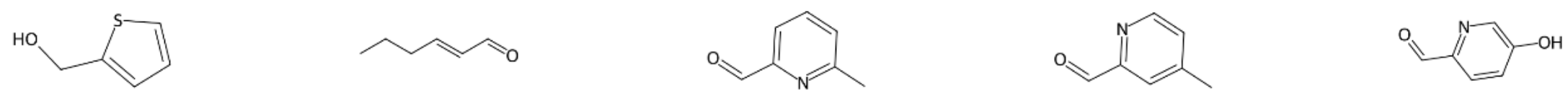

TA-115 TA-116 TA-117 TA-118 TA-119

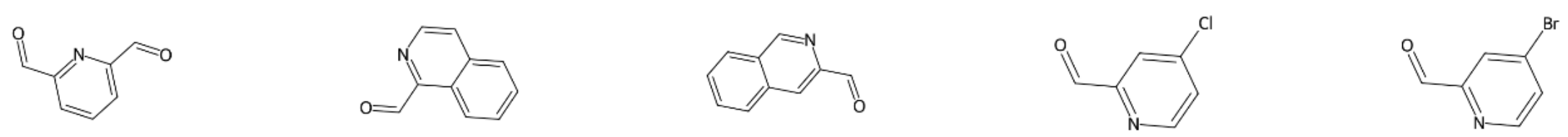

TA-120 TA-121 TA-122 TA-123 TA-124

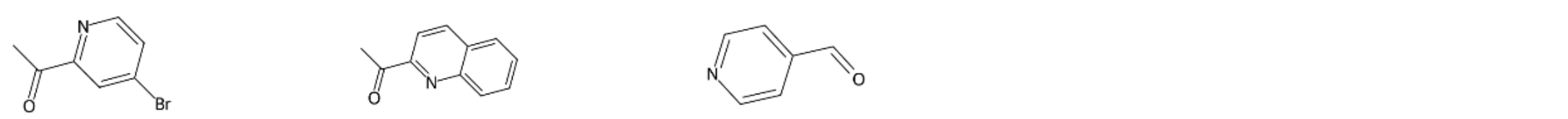

TA-125 TA-126 TA-127

Figure S2 (contd.): Substrates used for screening with transaminase enzymes.

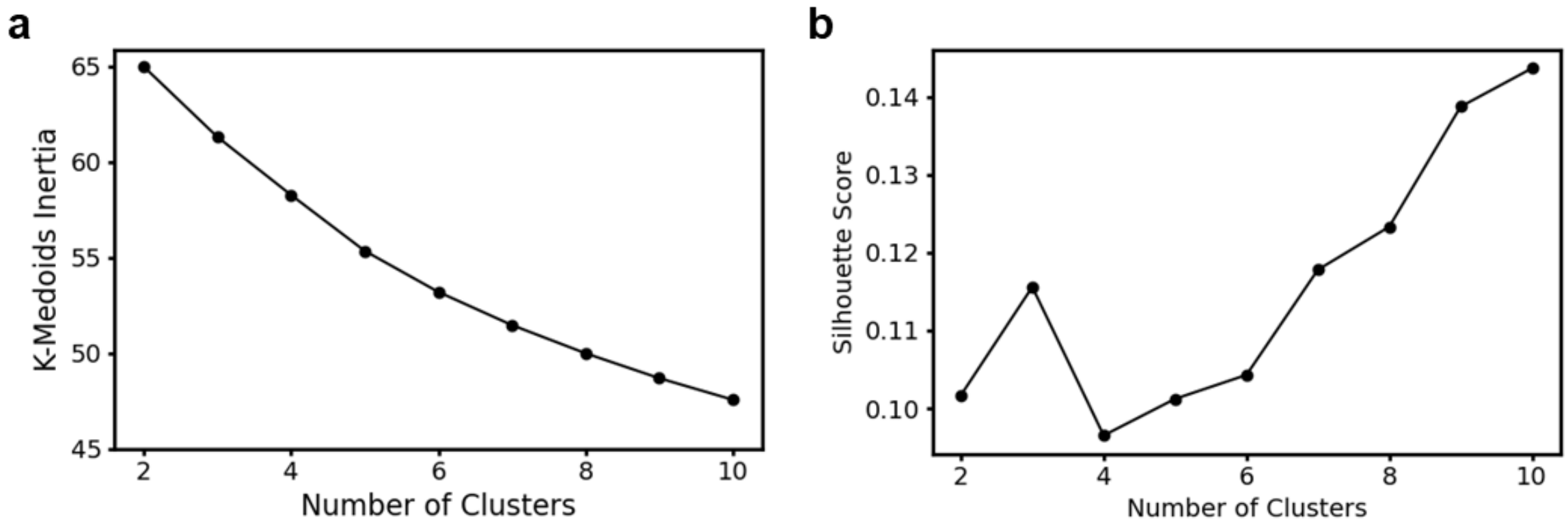

Figure S3: Clustering metrics and molecular diversity comparison for ADH substrates. **a,** k-Medoids total within-cluster inertia as a function of the number of clusters k, showing a monotonic decrease in average dissimilarity when partitioning into more groups. **b,** Corresponding average silhouette scores versus k, indicating modest improvements in cluster cohesion and separation—but remaining low (0.1–0.14), even at k = 10, consistent with overlapping chemical families.

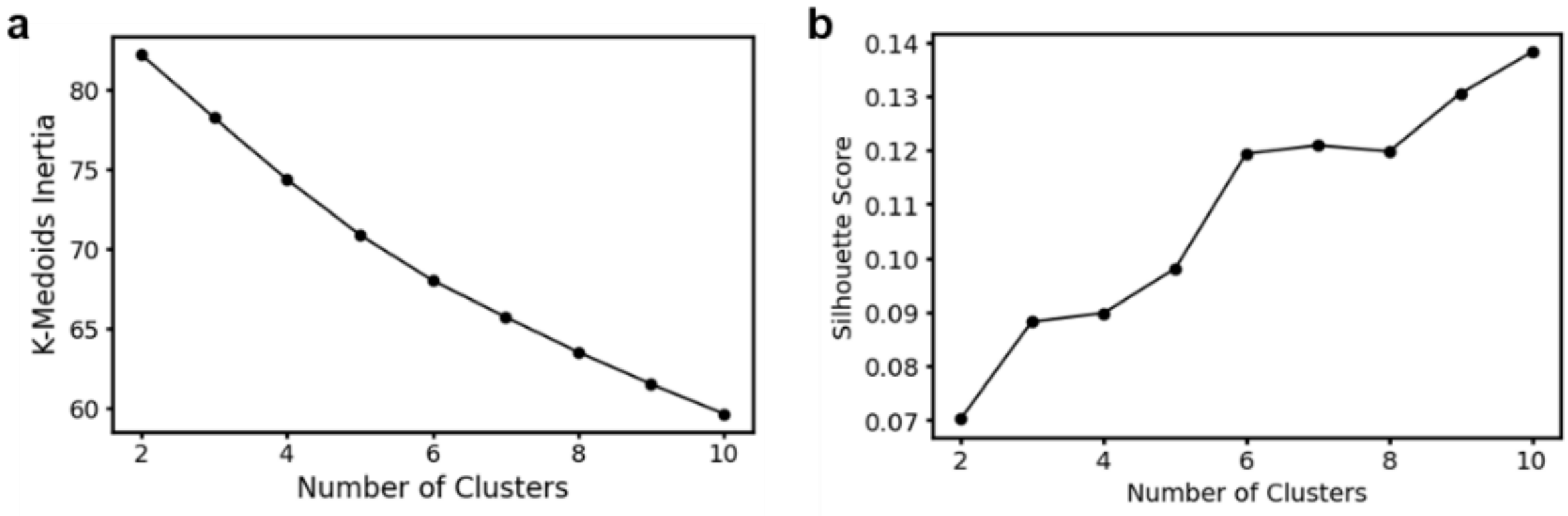

Figure S4: Clustering metrics and molecular diversity comparison for TA substrates. **a,** k-Medoids total within-cluster inertia as a function of the number of clusters k, showing a monotonic decrease in average dissimilarity when partitioning into more groups. **b,** Corresponding average silhouette scores versus k, indicating modest improvements in cluster cohesion and separation—but remaining low (0.07–0.14), even at k = 10, consistent with overlapping chemical families.

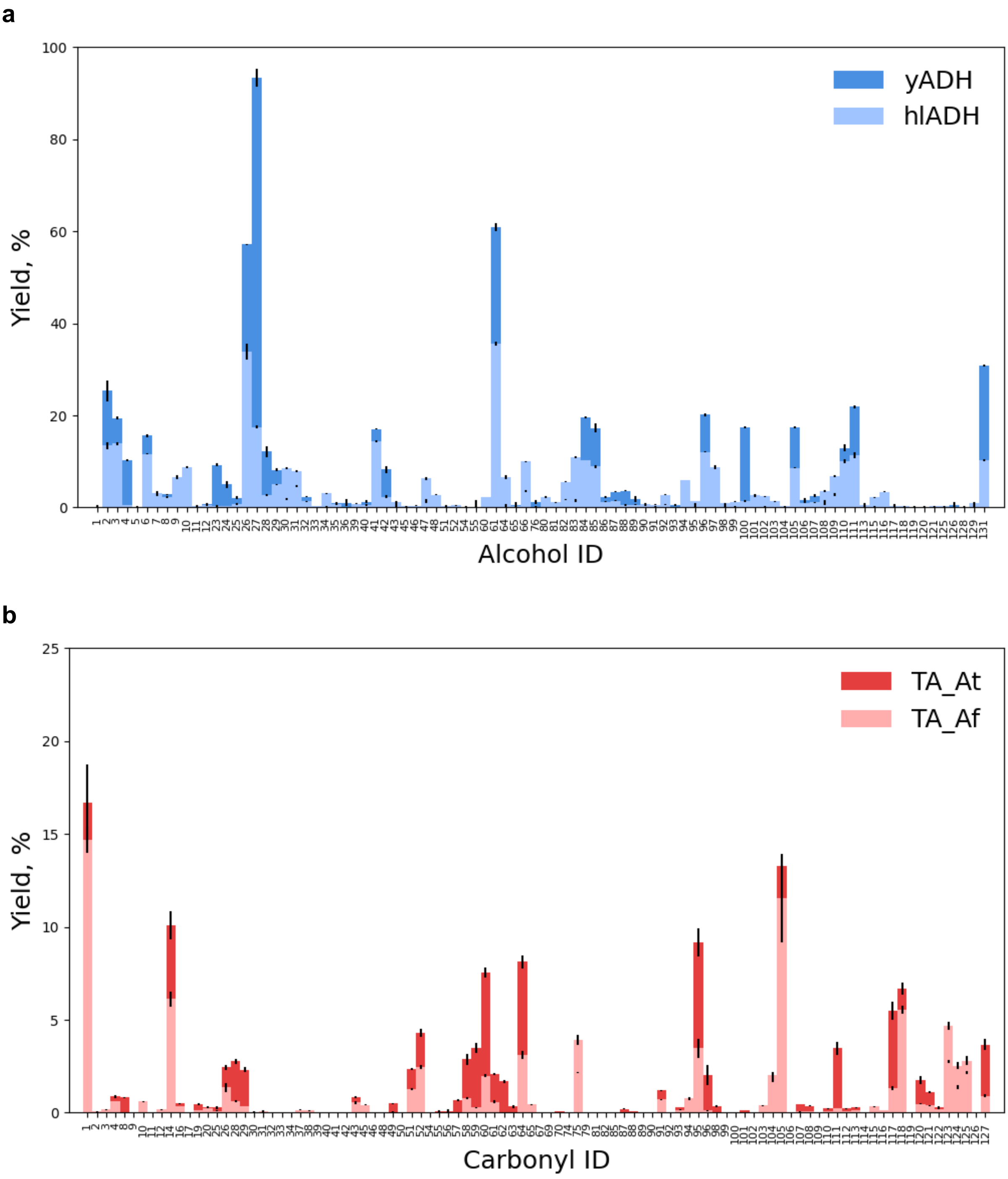

Figure S5: a, Histogram of reaction yields for alcohol oxidation of all substrates tested by yeast and horse liver ADHs. **b,** Histogram of reaction yields for transamination of carbonyl amine acceptors of all substrates tested by ω-transaminases from *A. terreus* and *A. fumigatus*. Standard deviations are reported for at least $n = 3$ replicates.

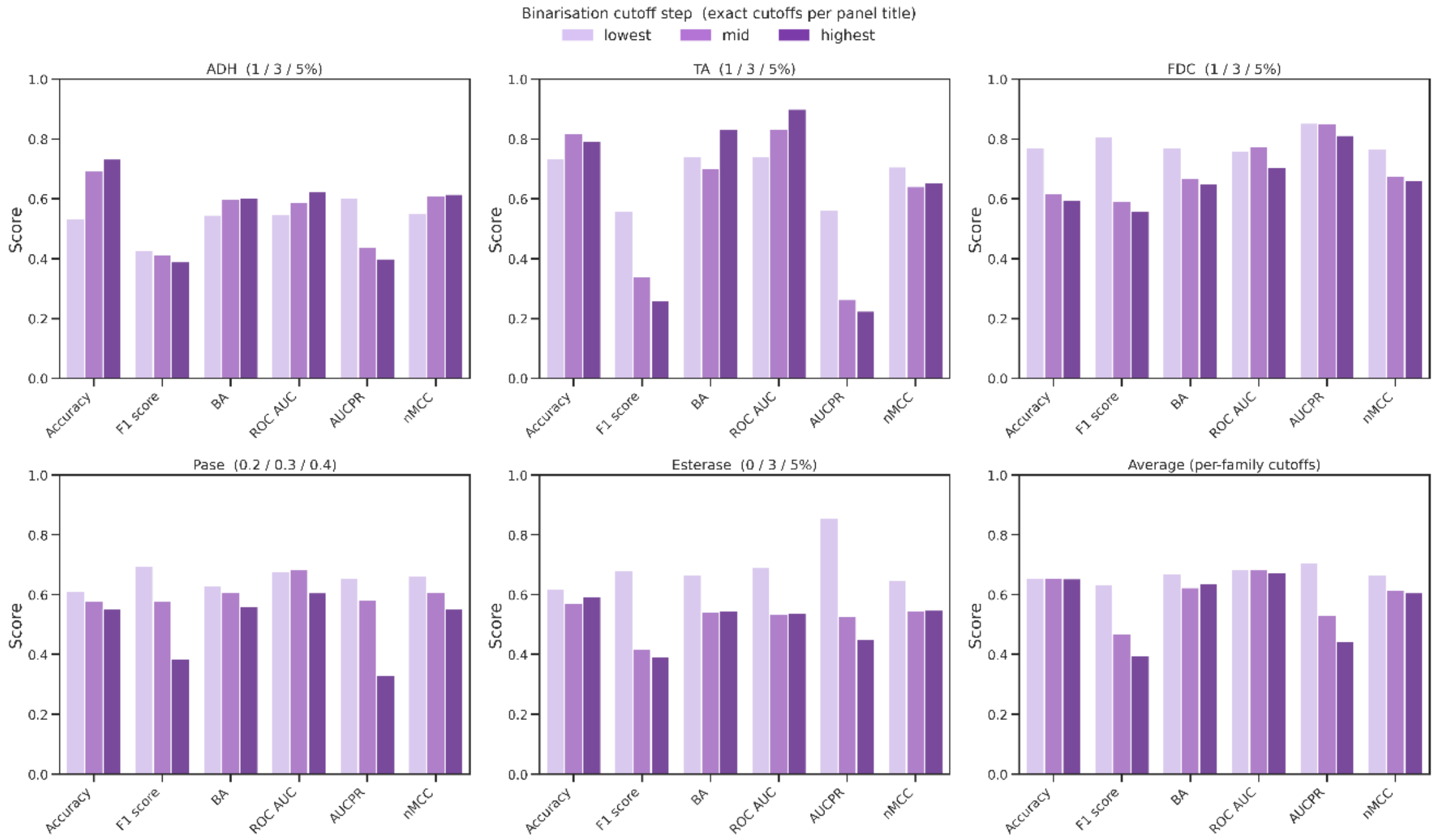

Figure S6. Performance of the RF model trained on the two TRAIN enzyme family datasets, validated on one VAL enzyme family, and tested on 2 TEST enzyme families. Three increasing active/inactive cutoffs (lightest to darkest bars) are applied to each of enzyme families: 1, 3, and 5% for ADH, TA and FDC; 0.2, 0.3 and 0.4 reactivity units for phosphatase; and activity > 0, 3% and 5% of the activity range for esterase. The first level reproduces the cutoffs used in the main text. Each panel reports six metrics (accuracy, F1, balanced accuracy, ROC-AUC, AUCPR, and normalized MCC = (1 + MCC)/2) at a fixed 0.5 decision threshold.

a)

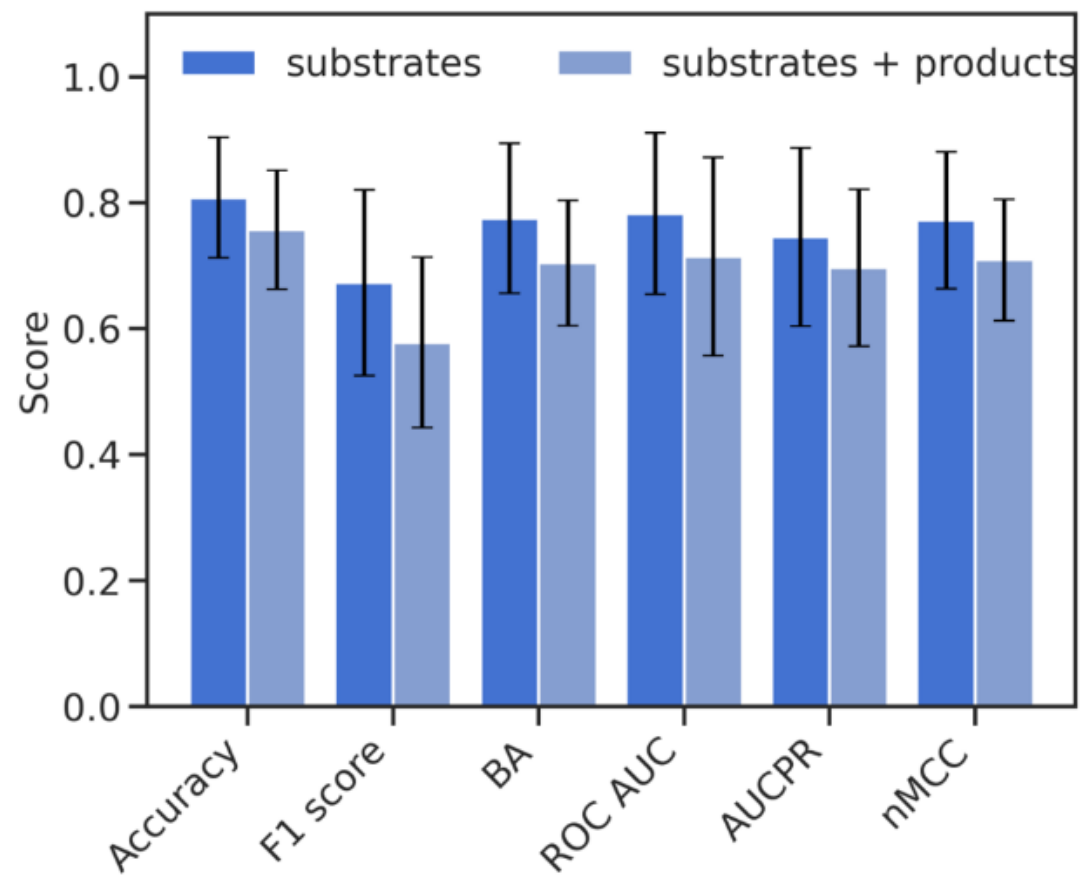

b)

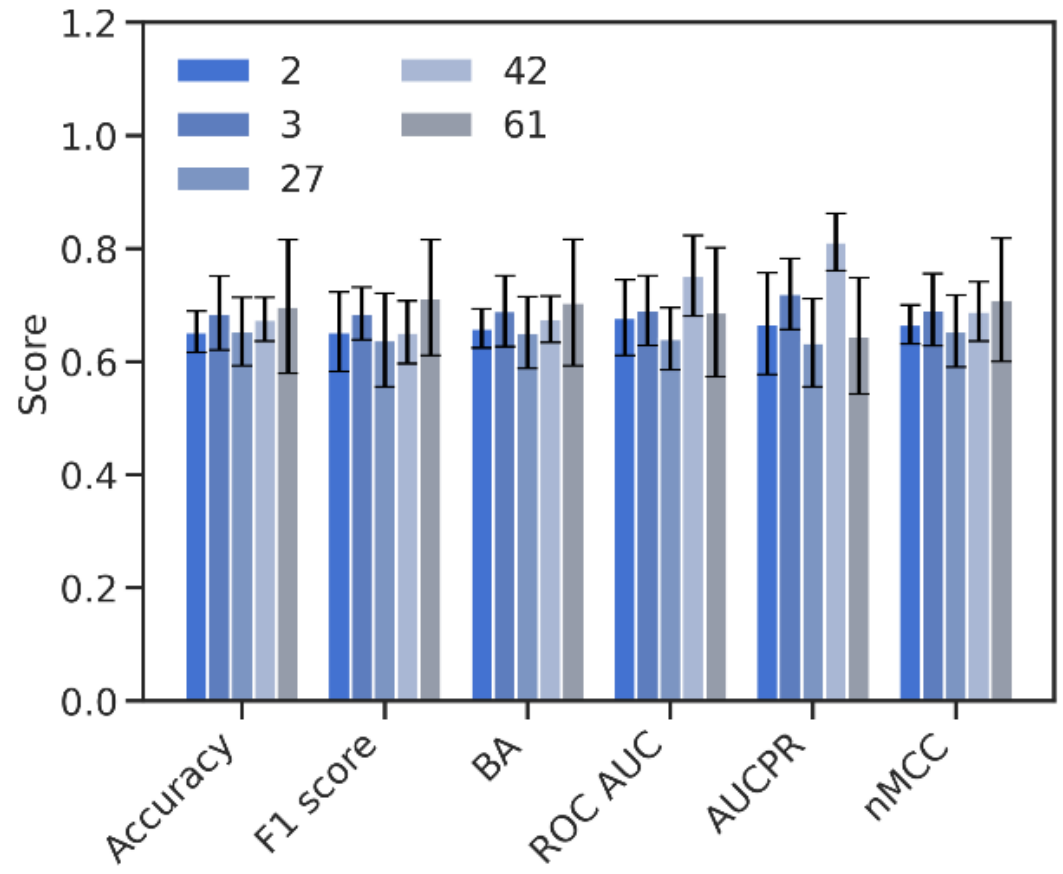

c)

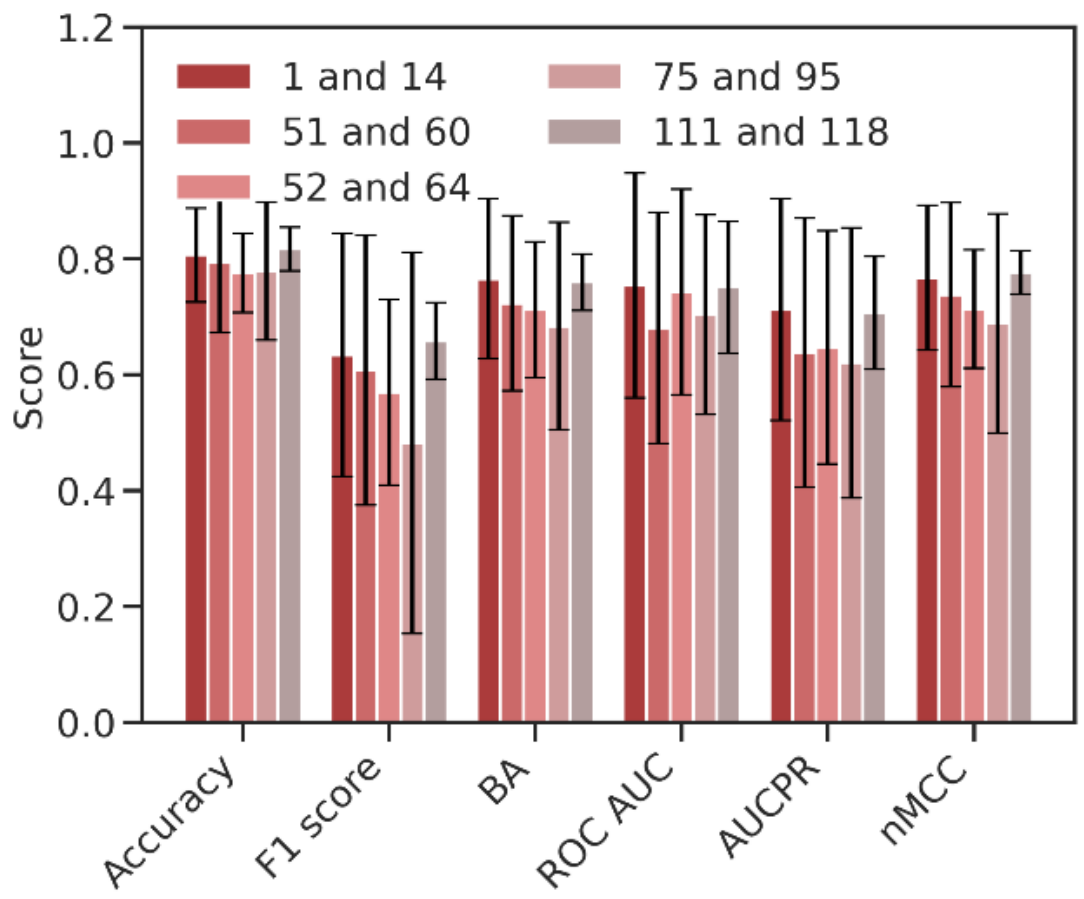

Figure S7: a, Performance of the random forest model trained and tested on the TA-At dataset using only substrate features (optimal five in the main text, dark blue bars) and using five substrate features plus features #3–6 of the corresponding products (light blue bars). Error bars indicate standard deviations from five-fold cross-validation. **b**, yADH dataset with compounds ADH-2, 3, 27, 42, and 61 with yields ranging from 8.3 to 93.3%; **c,** TA-At dataset with the following pairs of compounds used as reference substrates for ketones and aldehydes, respectively: 1 and 14, 51 and 60, 52 and 64, 75 and 95, and 111 and 118. The yields ranged from 2.1 to 16.7% for ketones and between 6.7 and 10.1% for aldehydes. See detailed yields of the compounds in Table S1.

a)

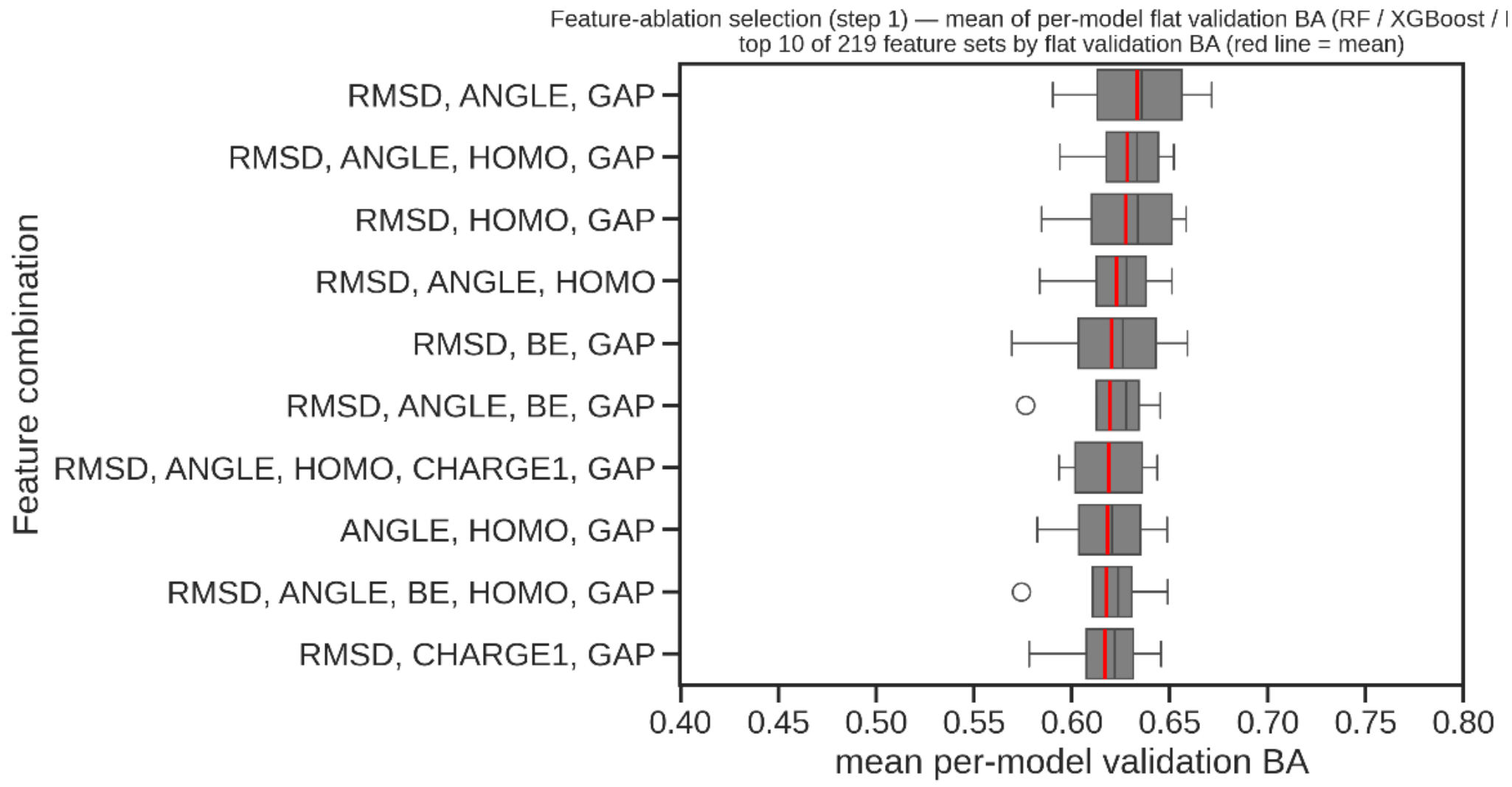

b)

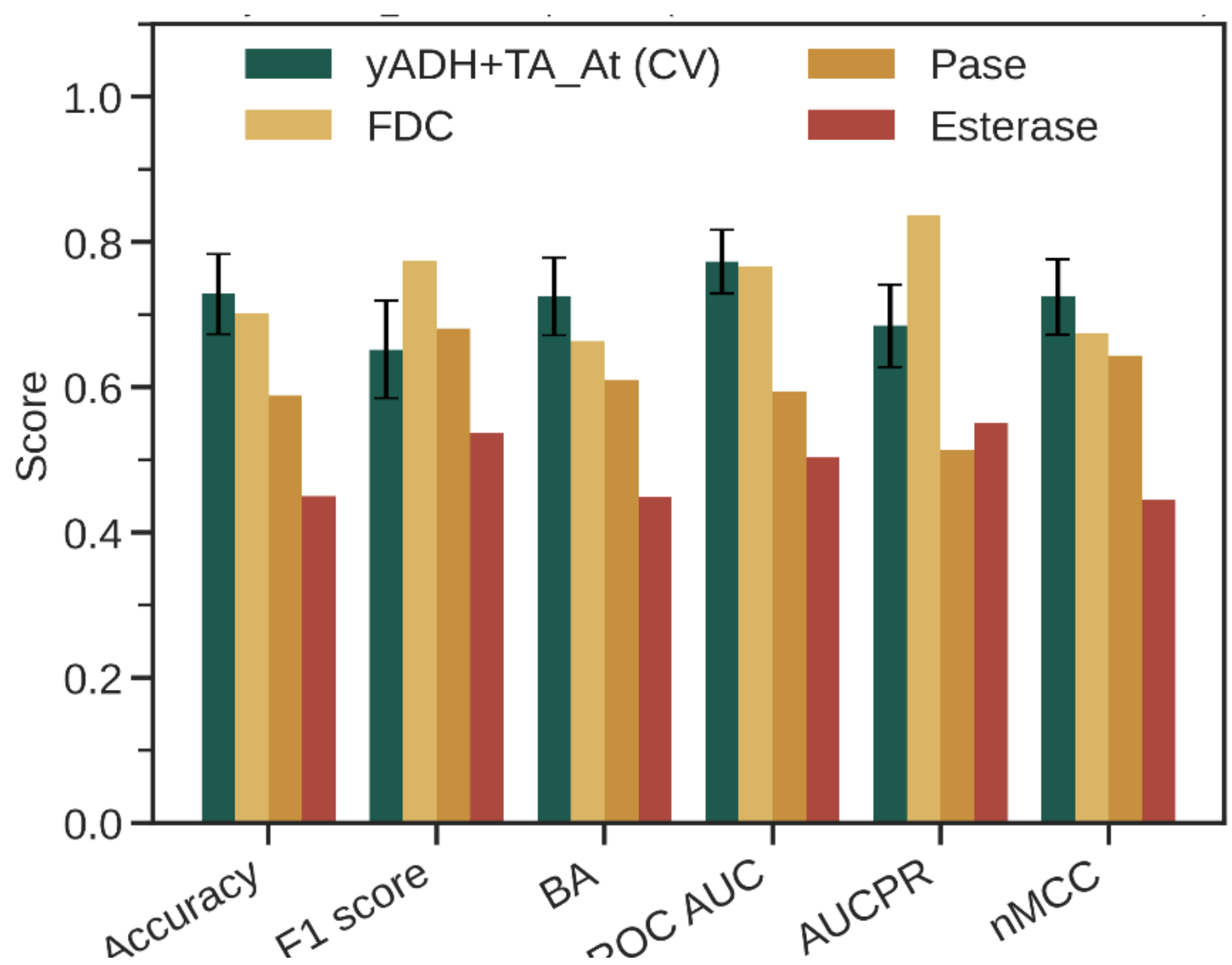

Figure S8: a, Top-10 feature combinations from the ablation study on eight features (see **section 2.5** in the main text) with 1–5 of features progressively removed for the random forests (RF) model trained on two enzyme family datasets and tested on the other two among ADH, TA, FDC, phosphatase (Pase), and esterase datasets, exhaustive combinations. The boxplot presents median and interquartile ranges of test balanced accuracy (BA) scores; the combinations are ranked by average BA value over all combinations. **b,** Performance of the RF model trained using five optimal features on 2 enzymes (yADH and TA-At) when tested on unseen literature data of substrates of fungal ferulic acid decarboxylases (FDCs, yellow bars) and *E. coli* phosphatase (pale orange bars), as well as ester hydrolase (esterase) CalB from *Pseudozyma aphidis* (dark orange bars).

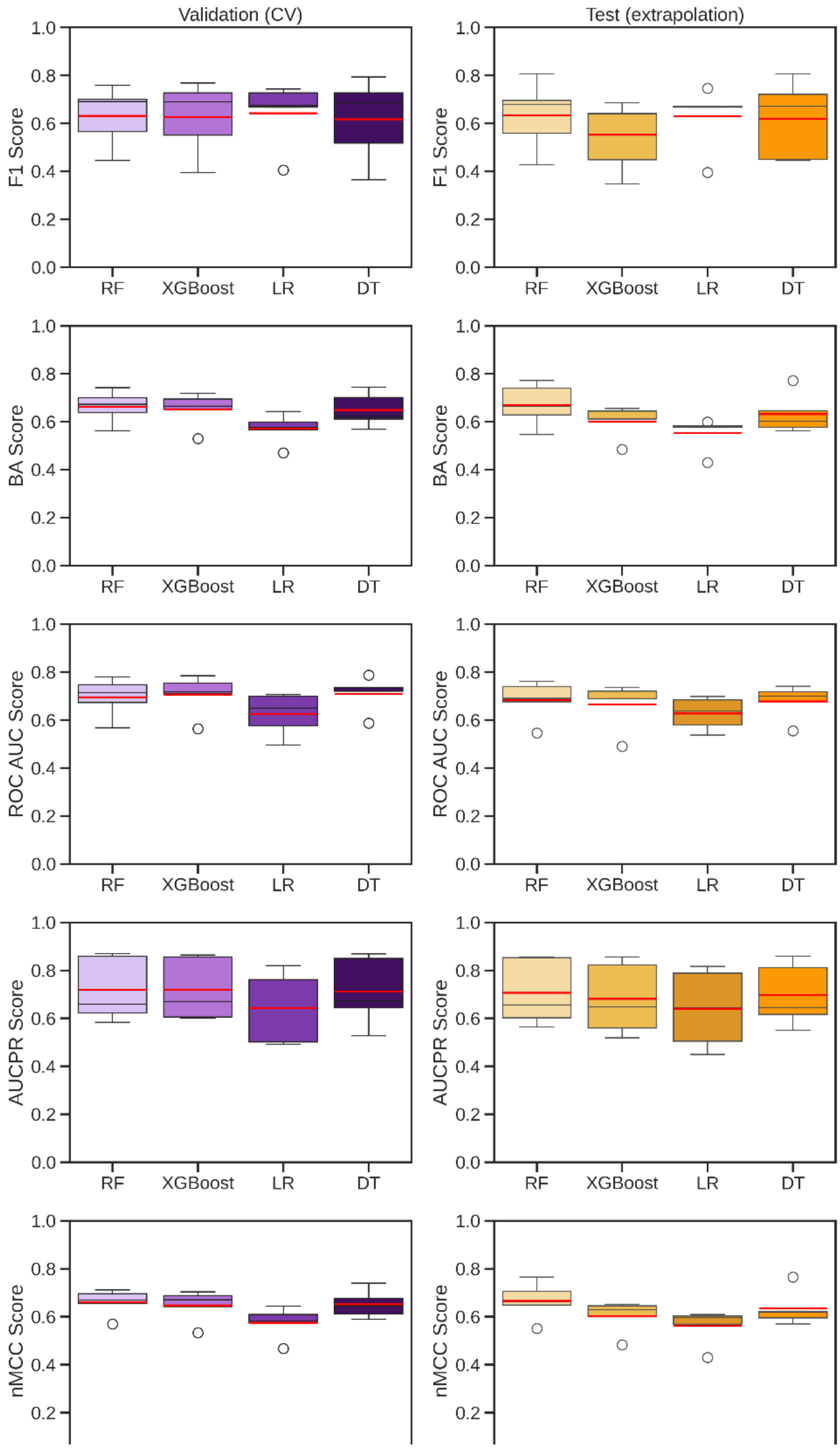

Figure S9: Model comparison and testing generalization abilities of logistic regression (LR), decision trees (DT), random forest (RF), and XGBoost (XGB) models using exhaustive combinations of datasets for five enzyme families: ADH, TA (this work), FDC[50], phosphatase[19,50], and esterase.[51] Panels show box plots, over 5 enzyme families with red/black lines indicating mean/median values respectively. First column shows VAL performance metrics and second column shows TEST performance metrics, that are following description in main text section 2.5 and supplementary 2.5. Each row shows F1 score, balanced accuracy, ROC AUC, AUCPR, nMCC scores respectively.

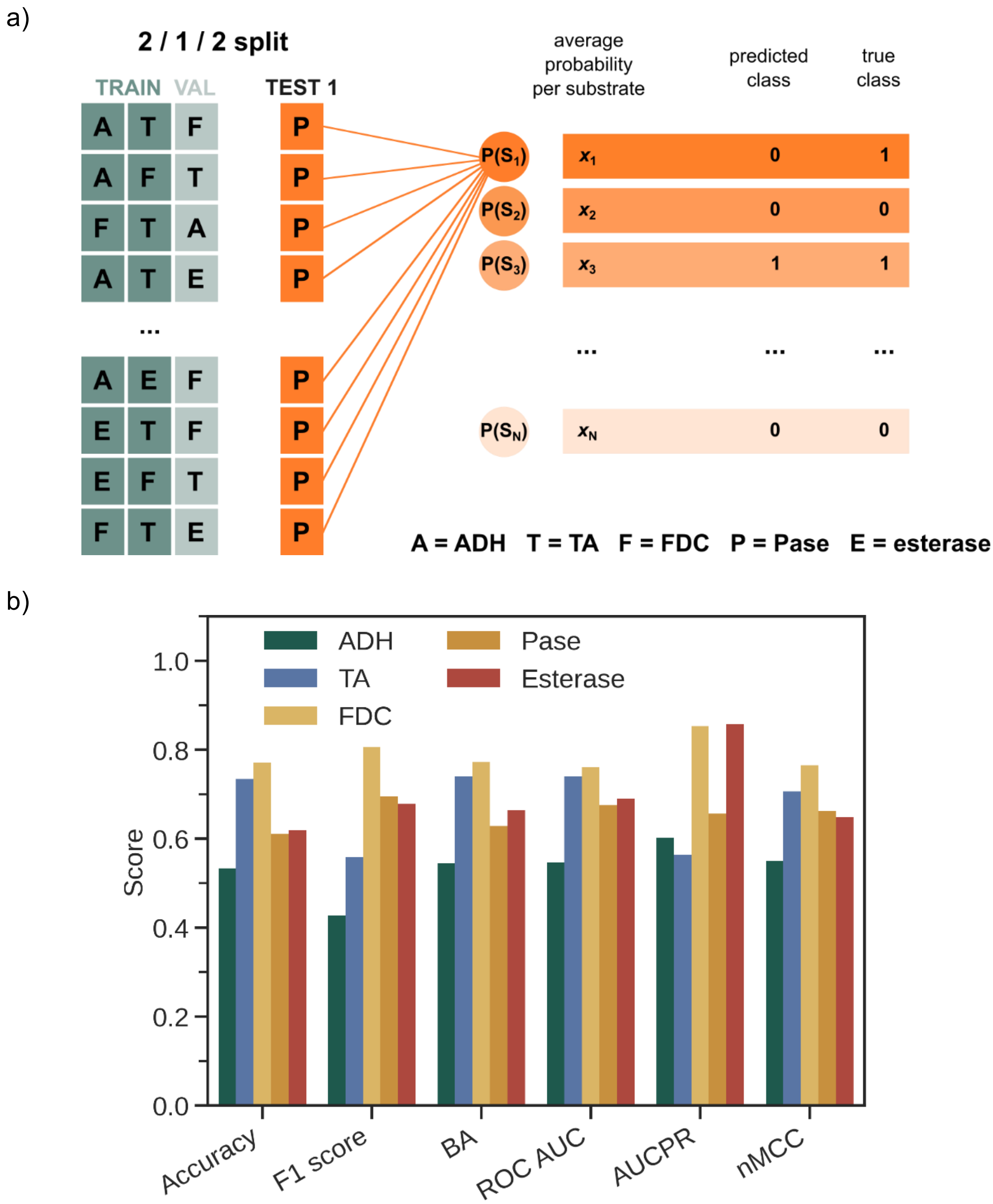

Figure S10: a, Scheme of exhaustive combination splits of 2 TRAIN / 1 VAL / 2 TEST for 5 enzyme family datasets. **b**, optimized RF three-feature based model performance family-wise.

a)

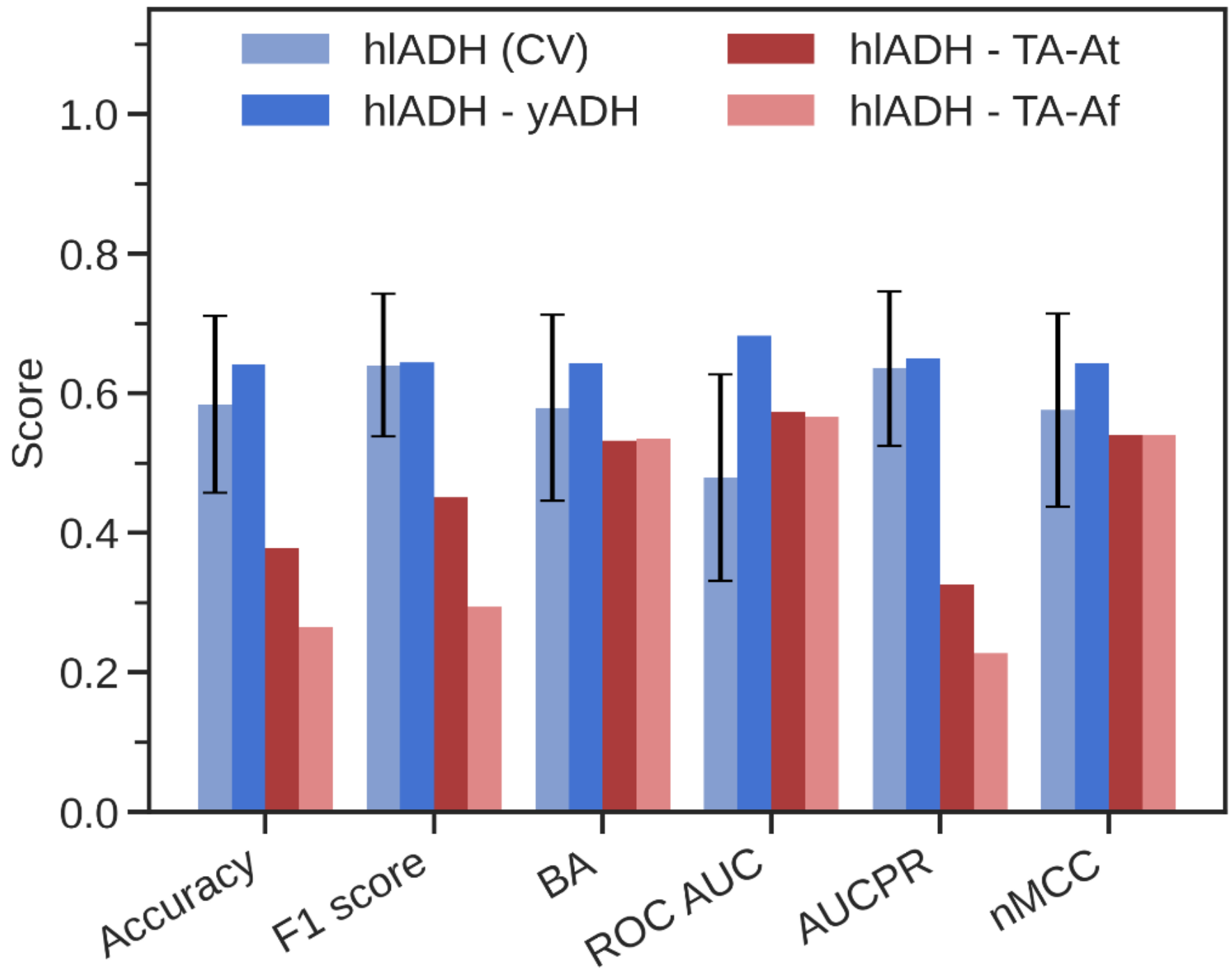

b)

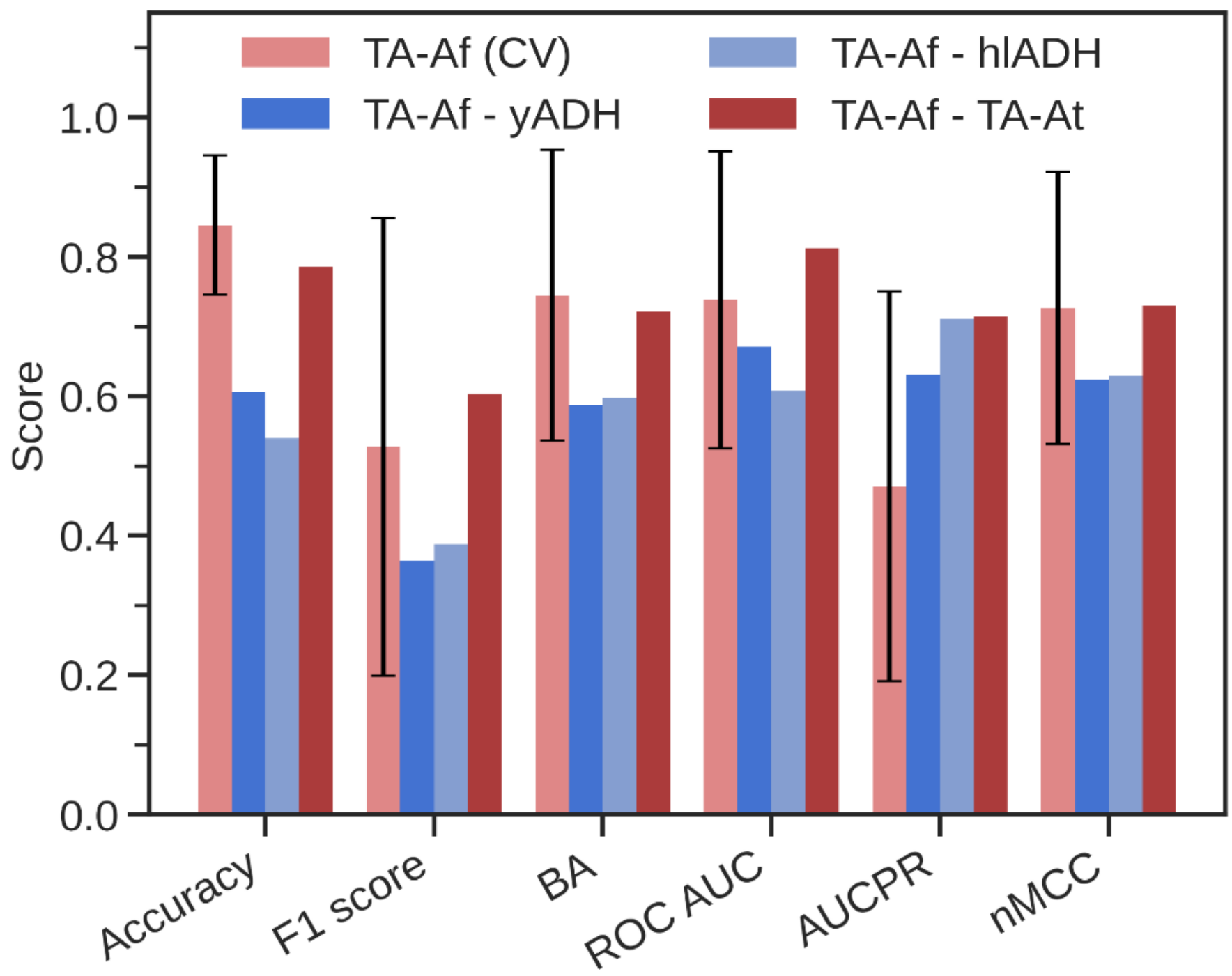

Figure S11: **a**, Random forest model trained on hlADH data and tested on three other enzyme datasets (yADH, TA-At, or TA-Af) as unseen. **b**, Random forest model trained on ω-TA-Af and tested on three other enzyme datasets (yADH, hlADH, or TA-At) as unseen. Error bars for training and test sets indicate standard deviations from 5-fold CV.

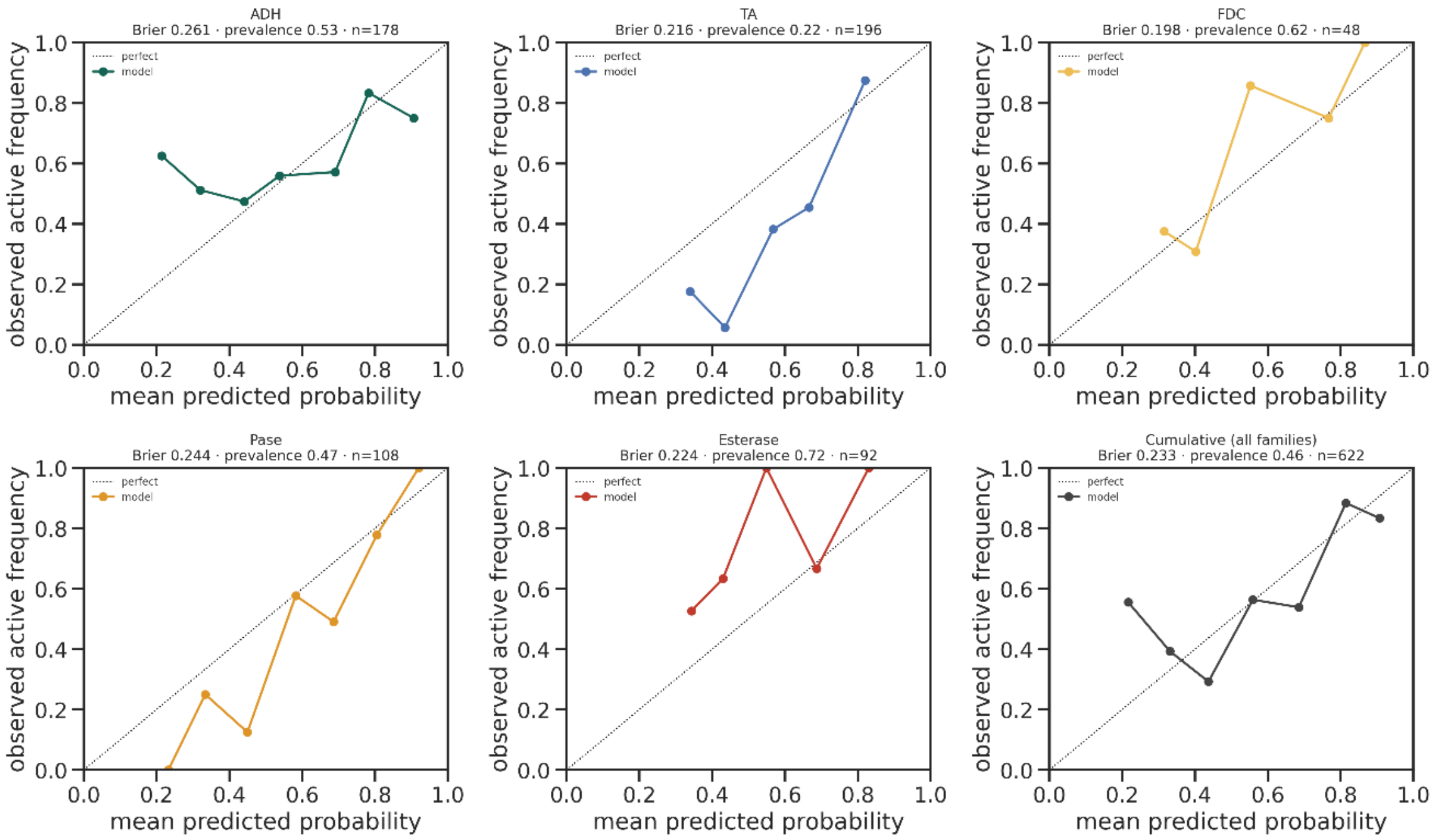

Figure S12. Calibration curves for the five enzyme family prediction models. Panels: ADH (n = 178), TA (n = 196), FDC (n = 48), phosphatase (n = 108), esterase (n = 92) and all families pooled (n = 622). The curve represents how the frequency of true positives (actives) correlates with the mean predicted probability, with the diagonal denoting perfect calibration. Calibration curves are characterized with Brier scores and prevalence. The Brier score is the mean squared difference between the predicted active-class probability and the observed binary outcome (1 for active, 0 for inactive), averaged over all substrates of a family; it ranges from 0 for perfect probabilistic agreement to 1 for maximal disagreement, so lower values denote better-calibrated probabilities; the prevalence is the proportion of substrates in a family that are active, i.e. the positive-class base rate; it sets the reference level for the Brier score, since a non-informative model that assigns the prevalence to every substrate attains a Brier score of prevalence × (1 − prevalence). Models shown here have the calibration curve lying near the diagonal, indicating that the predicted probabilities match the observed active frequencies, with Brier score in 0.199–0.254 (0.230 pooled). It indicates that our predicted probabilities are usable as calibrated confidences, e.g., higher scores correspond to higher confidence of prediction.

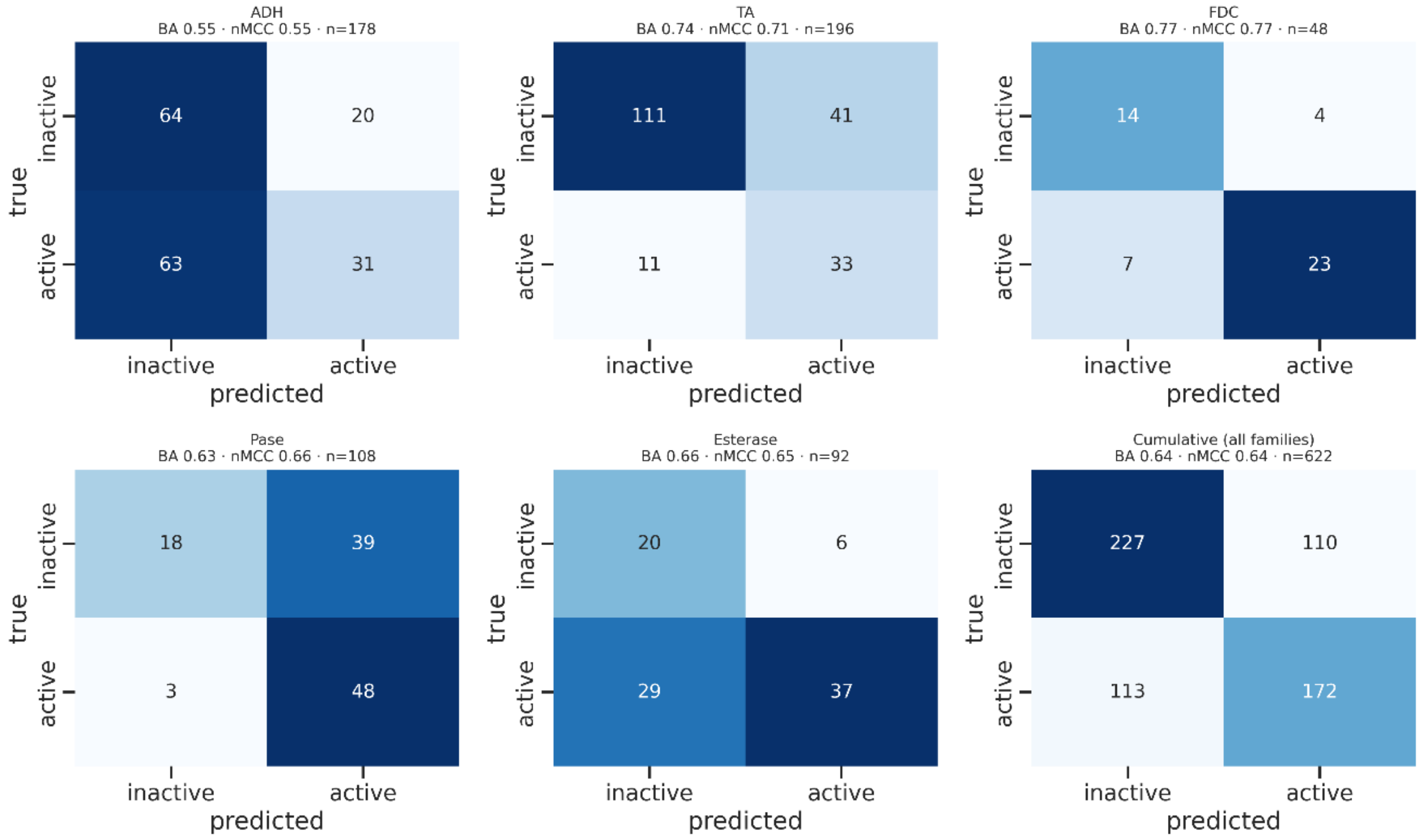

Figure S13. Per-family confusion matrices for the five enzyme family prediction models. Confusion matrices (rows, true class; columns, predicted class) at the fixed 0.5 decision threshold. Panels: ADH, TA, FDC, phosphatase, esterase, and all families pooled. Each title gives the BA, nMCC, and the number of substrates. nMCC exceeds 0.5 (chance) in every family (0.58 to 0.77; 0.65 pooled) but has different prevalence on prediction of active or inactive molecules.

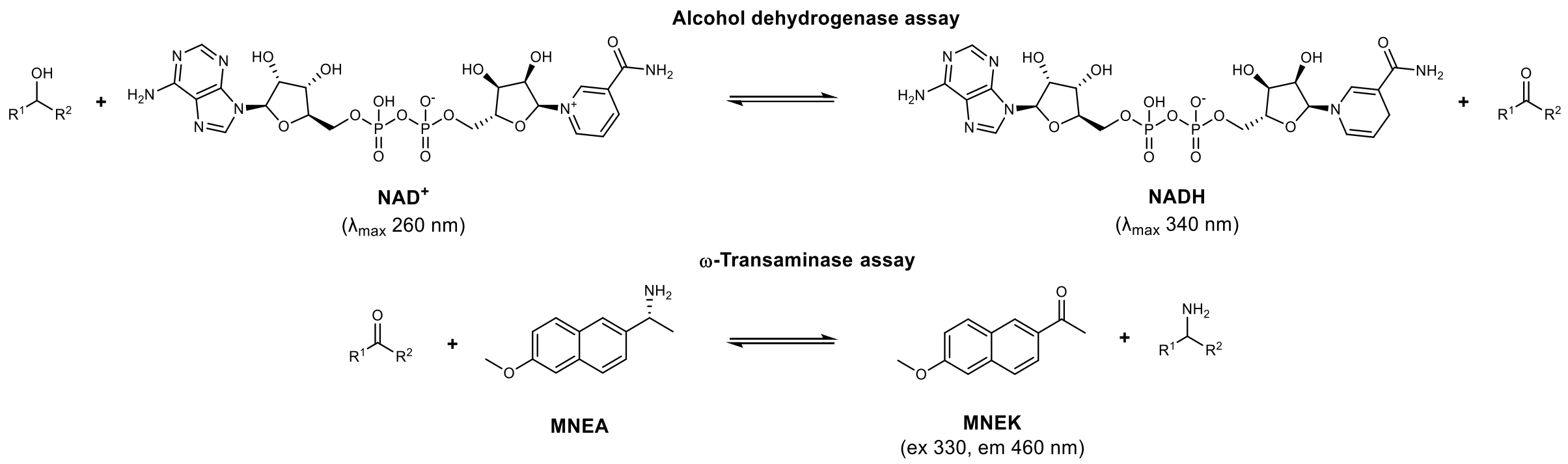

Figure S14: Assays used to evaluate enzyme activity. (top) Alcohol dehydrogenase (ADH) activity was assessed using nicotinamide adenine dinucleotide (NAD^+), the enzyme's cofactor, as the assay analyte. (bottom) ω-Transaminase (TA) activity was measured using 1-(6-methoxynaphth-2-yl)ethylamine (MNEA) as the co-substrate producing fluorescent ketone used to follow the reaction.

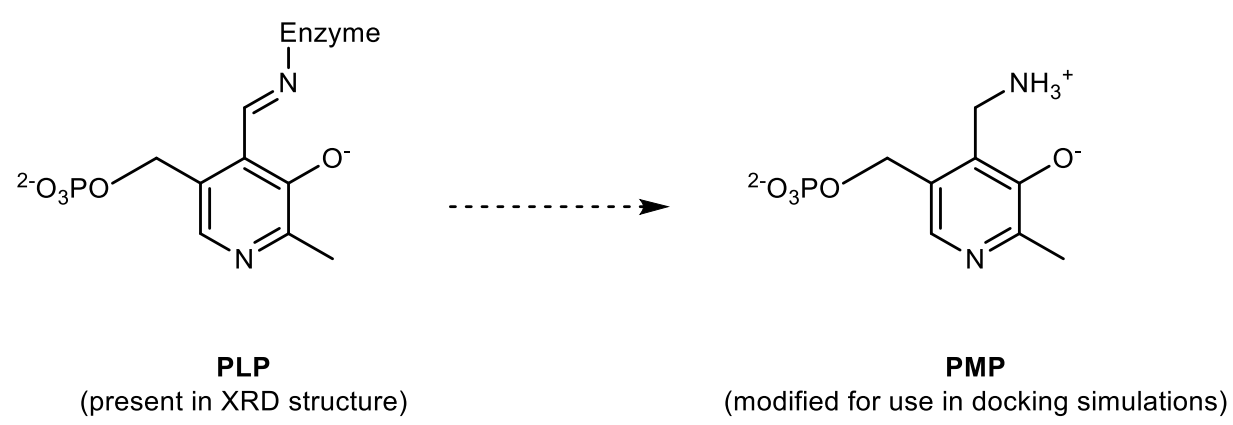

Figure S15: Pyridoxal phosphate cofactors in the active site of ω-transaminases. (left) Pyridoxal 5'-phosphate (PLP), covalently bound to the enzyme via a Schiff base linkage, as present in the downloaded crystal structures (PDB IDs 4CE5 and 4CHI). (Right) Pyridoxamine 5'-phosphate (PMP), generated by in silico modification of PLP, used for docking simulations.

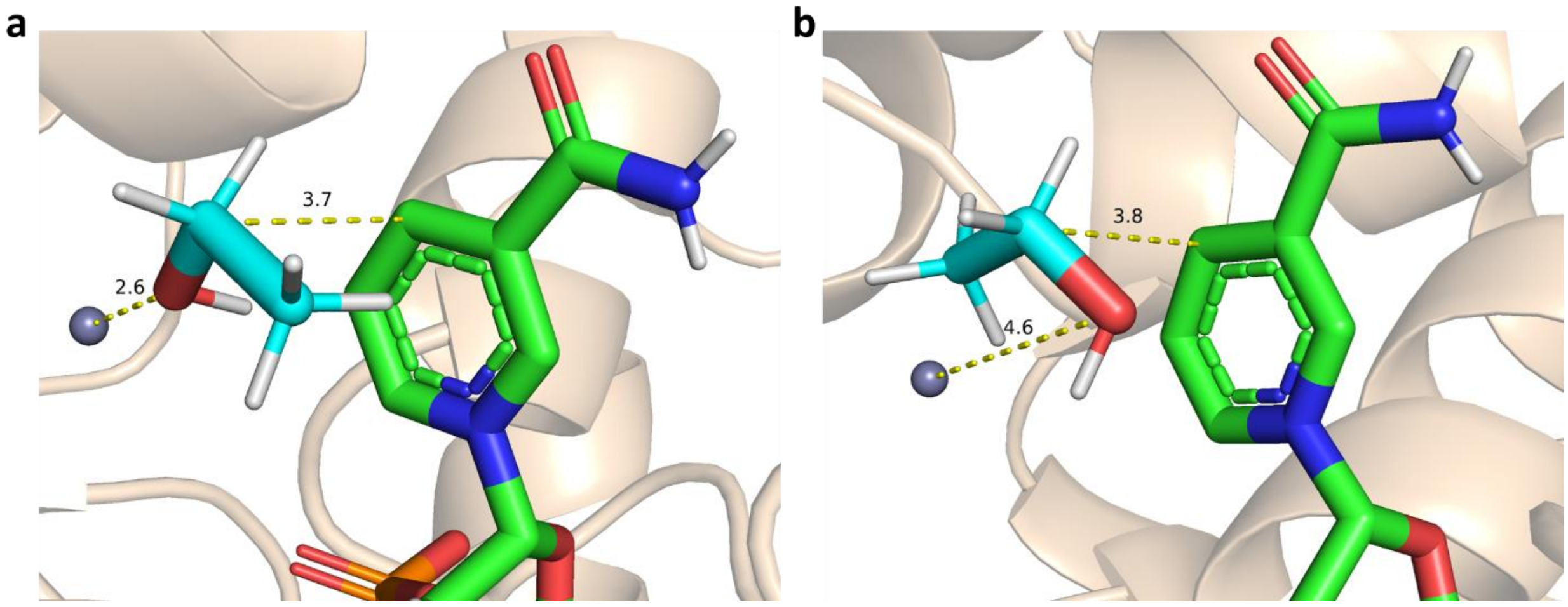

Figure S16. a, Docked pose of the reference substrate ethanol (cyan) in the yADH active site (PDB ID 7KCB). The hydroxyl oxygen coordinates the catalytic Zn^{2+} (grey sphere; O–Zn = 2.6 Å) and the hydride-donating α-carbon is directed toward C4 of the NAD^+ nicotinamide ring (green; Cα–C4 = 3.7 Å), consistent with a catalytically productive geometry. b, Docked pose of the reference substrate ethanol (cyan) in the hlADH active site (PDB ID 2OHX). The hydride-donating α-carbon is aligned with C4 of the NAD^+ nicotinamide ring (green; Cα–C4 = 3.8 Å), with the hydroxyl oriented toward the catalytic Zn^{2+} (grey sphere; 4.6 Å), consistent with a catalytically productive geometry. Selected distances are shown in Å.

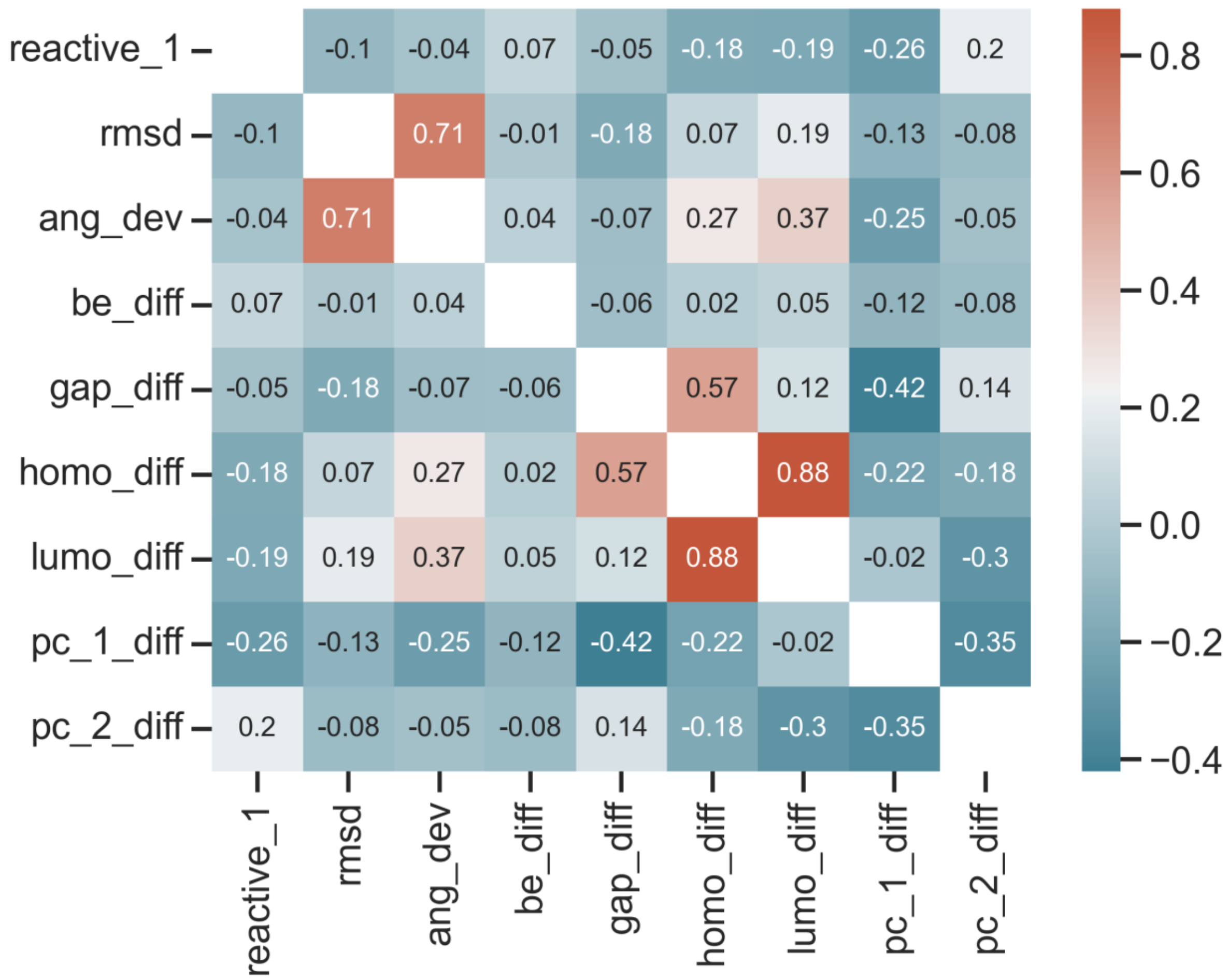

Figure S17: Correlation matrix of eight relative (candidate substrate vs reference) features derived from docking (rmsd, RMSD; ang_dev, angular deviation; be_diff, binding energy difference; gap_diff, HOMO–LUMO gap difference; homo_diff, HOMO energy difference; lumo_diff, LUMO energy difference; pc_1_diff, partial charge 1 difference; and pc_2_diff, partial charge 2 difference).
