# Supplementary material for: Robots and Minimal, Physics‐Informed Features: A Hybrid Framework for Enzyme Catalysis
Source: Adv Sci (Weinh). 2026 Sep 11:e77474. Online ahead of print. doi: 10.1002/advs.77474 (PMC13564181; doi:10.1002/advs.77474)
Supplement: Supplementary file 2 [file ADVS-9999-e77474-s004.docx]

**Table S1** provides enzyme activity datasets used in the paper, both obtained in the current work (yADH, hlADH, TA-At, and TA-Af) and extracted from literature (FDC, phosphatase, and esterase). In particular, experimental yields, docking- (RMSD of headgroup atoms, angular deviation, binding energy difference with respect to reference compounds) and QM-derived features (HOMO, LUMO energy differences and the gap difference, and partial charges of the atoms of the headgroup) are reported. Next, the table reports hyperparameter grids used to optimize the ML models. Finally, model performance metrics used to plot **Figures 3a**, **4**, **5**, and **6** of the main text, as well as **Figures S6-S8**, **S10b**, and **S11-S13** in the **SI**, are reported.
